# FSD-Net: underwater object detection based on frequency and spatial domain feature enhancement

**DOI:** 10.3389/frai.2026.1770342

**Published:** 2026-04-17

**Authors:** Chao Zhang, Shuang Wu, Baohua Huang, Binchen Zhao, Fengqi Cui, Xingkun Li

**Affiliations:** 1 College of Transport Geography, Shandong Jiaotong University, Jinan, China; 2 College of Land Resources and Surveying Engineering, Shandong Agriculture and Engineering University, Jinan, China; 3 School of Physical Sciences, Qingdao University, Qingdao, China

**Keywords:** computer vision, deep learning, feature enhancement, frequency domain, underwater object detection

## Abstract

**Background:**

Complex underwater visual conditions cause severe missed and false detections in conventional object detection models, hindering reliable autonomous underwater exploration. This work addresses these key performance limitations.

**Methods:**

We propose FSD-Net, a novel underwater detection model with two core enhancement modules. The Frequency Attention Convolution Module reduces missed detections via frequency-domain spatial feature preservation, and the Multi-dimensional Feature Enhancement Module suppresses false detections via enhanced semantic fusion. Experiments include ablation studies and state-of-the-art method comparisons on the UTDAC2020 and Brackish datasets.

**Results:**

FSD-Net achieves state-of-the-art performance on both tested datasets. On the UTDAC2020 dataset, it reaches 85.7% AP50 and 82.5% F1-score, with a 3.8% AP50 improvement over the baseline model. On the Brackish dataset, it achieves 98.1% AP50 and 97.0% F1-score, with a 3.9% AP50 improvement over the baseline. The model outperforms all compared mainstream detection algorithms, and ablation studies validate the effectiveness of both proposed modules.

**Conclusion:**

FSD-Net's joint frequency-spatial enhancement strategy effectively mitigates underwater image degradation challenges, providing a robust detection solution for autonomous underwater exploration. The proposed dual-module design offers a practical reference for detection model optimization in complex visual environments, with future work focused on lightweight model optimization.

## 1 Introduction

**Figure 1:**
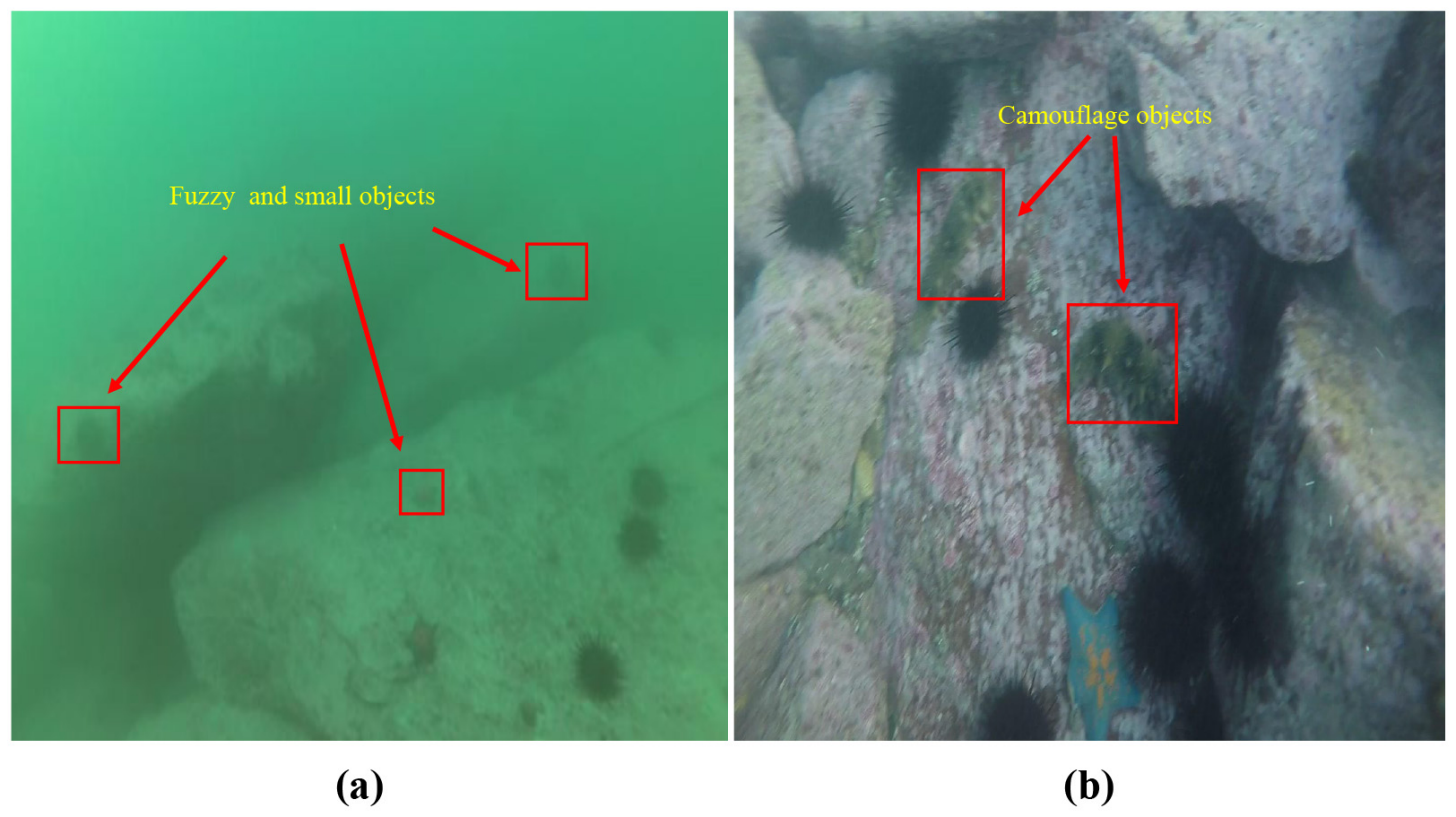
Challenges in underwater object detection: examples of difficult targets. **(a)** shows image blur and small targets in underwater environments; **(b)** presents the protective coloration of marine organisms highly indistinguishable from the background. Both scenarios represent the major challenges for underwater target detection.

**Figure 2:**
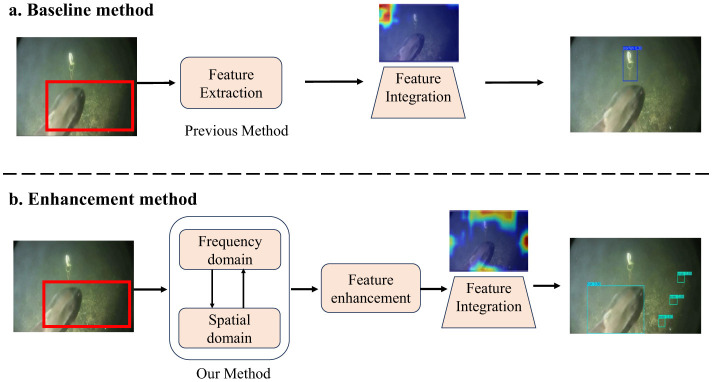
Previous methods rely on convolutional operations for local feature extraction, which leads to the degradation of shallow spatial features as the network depth increases. This misalignment with deep semantic features often results in missed detections. Moreover, due to underwater noise interference, inaccurate fusion of deep channel semantic features can cause false positives. Our approach effectively preserves global semantic information through frequency domain transformations and re-enhances deep features, thereby improving the perception and detection of underwater targets.

Rich marine resources play a crucial role in addressing global energy demands and promoting economic development (Gao J. et al., 2024; Hua et al., 2023). As a result, ocean exploration has become a prominent research focus. However, traditional detection methods suffer from high computational costs and rely heavily on manual parameter tuning, making them inadequate for meeting growing application demands (Chen et al., 2024). In addition, the low efficiency and inherent risks of manned underwater exploration limit its ability to support large-scale ocean investigation (Jian et al., 2024; Fu et al., 2023). Research on unmanned detection technologies is essential for enabling efficient and scalable ocean exploration. Conventional underwater detection methods rely on handcrafted descriptors that extract a limited set of features to detect and localize underwater targets. These descriptors are designed to capture specific underwater object characteristics, such as contours and edges, but they struggle to adapt to the complex and dynamic marine environments (Fu et al., 2023). The advent of Convolutional Neural Networks (CNNs) has revolutionized computer vision by enabling automatic feature extraction (Jin et al., 2022). Specifically, CNN-based object detection models, including two-stage methods like Faster R-CNN (Ren et al., 2016) and one-stage approaches such as YOLO and EfficientDet, have significantly advanced the field (Chen et al., 2022). However, due to the complexity of underwater environments, directly applying deep learning models to underwater detection often results in performance degradation (Wang et al., 2023). The challenges of underwater detection are illustrated in Figure 1a, underwater targets are often small and blurry. This makes it difficult for models to extract meaningful semantic features. As shown in Figure 1b, many underwater organisms exhibit camouflage that closely resembles the background, making it difficult for models to distinguish between foreground and background (Guo et al., 2024). These challenges lead to false positives and missed detections by the detection model, as exemplified in Figure 2a.

To address the challenges associated with object detection in underwater images, this study proposes a novel network that includes two methods: the Multi-dimensional Feature Enhancement Module (MFEM) and the Frequency Attention Convolution Module (FACM). Firstly, to mitigate the loss of shallow spatial contour information and enhance the alignment between low-level and high-level features within the backbone network, the Frequency Attention Convolution Module (FACM) is introduced. FACM leverages the Fourier transform to convert the feature maps extracted by the convolutional module from the spatial domain to the frequency domain. By alternating between frequency and spatial domains, the method preserves low-level global spatial information, facilitating the integration of spatial details and channel semantics in deep networks. This ultimately improves localization accuracy and mitigates missed detections in underwater environments. Secondly, underwater noise corruption degrades the accuracy of deep semantic features, while multi-scale object imbalance leads to detection failures. To address these challenges, we propose the Multi-dimensional Feature Enhancement Module (MFEM), which improves target perception robustness through adaptive semantic fusion. MFEM enhances underwater target features by applying parallel weighting across spatial and channel dimensions, followed by residual addition and channel concatenation to further strengthen feature representation. The resulting weighted features emphasize effective foreground information while suppressing noise and background interference.

**Figure 3:**
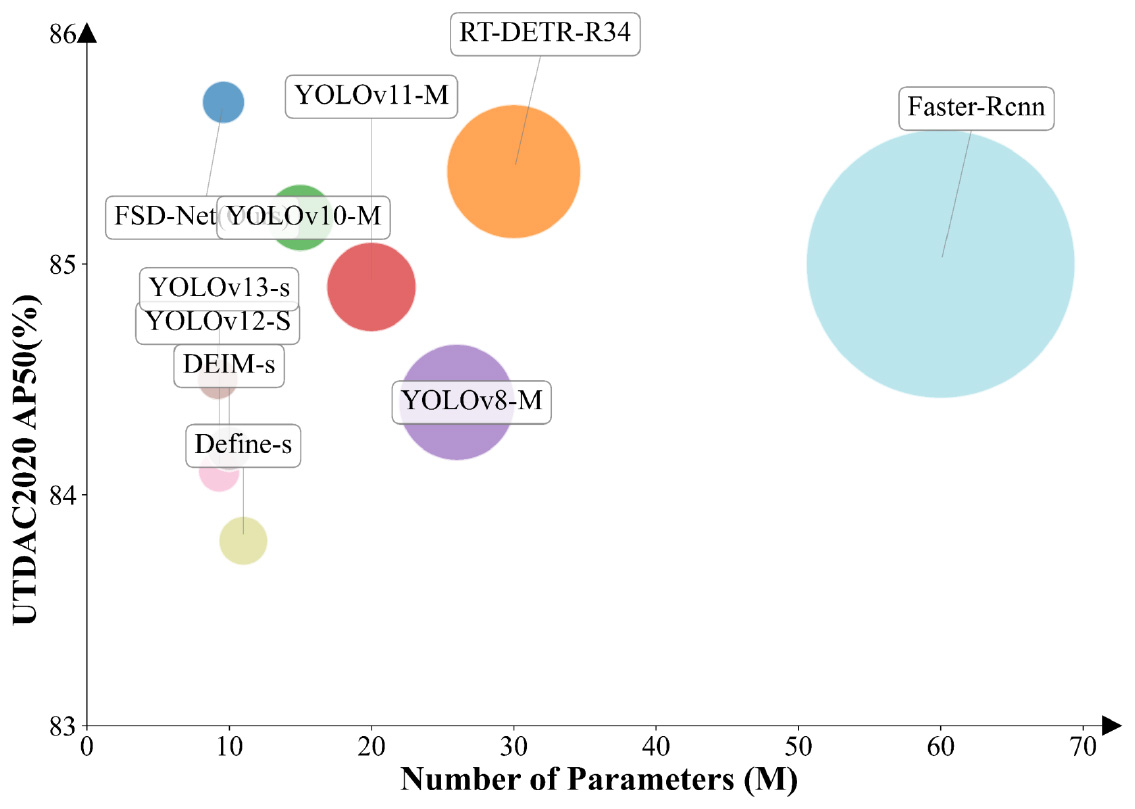
FSD-Net is compared with other detectors in terms of accuracy on UTDAC2020 dataset. The radius of the circle represents GFLOPs.

To enable accurate underwater exploration, we propose FSD-Net, a novel detection framework that significantly outperforms the baseline model YOLOv11s in terms of underwater object detection precision. Extensive experiments conducted on the UTDAC2020 and Brackish datasets demonstrate that our proposed detector achieves state-of-the-art performance in complex underwater environments with strong interference, as illustrated in Figure 3. The core innovation of FSD-Net lies in constructing an inherent enhancement mechanism inside the feature learning process, rather than applying additional post-hoc operations or standalone enhancement branches. By embedding the FACM and MFEM units into the intermediate feature hierarchy, the model simultaneously performs low-level spatial-semantic alignment, high-frequency noise suppression, and multi-dimensional feature weighting during feature extraction, without requiring any offline image enhancement, extra data pre-processing, or decoupled optimization stages. In this way, FSD-Net breaks through the conventional “separate enhancement + separate detection” pipeline and establishes a new paradigm: enhancement is no longer an auxiliary task, but becomes an intrinsic part of feature representation learning. This paradigm not only ensures that the entire model is optimized coherently for the final detection objective but also avoids information loss and domain gap caused by independent enhancement strategies. Therefore, the contribution of FSD-Net is not limited to module-level combination, but provides a new principled paradigm for underwater object detection: one-stage, end-to-end, feature-domain inherent enhancement unified with detection.

The contributions of this study can be summarized as follows.

- This study proposes FSD-Net, a novel underwater object detector that achieves precise target localization. Extensive experiments conducted on the UTDAC2020 and Brackish datasets demonstrate its superior detection accuracy, surpassing existing state-of-the-art methods while maintaining competitive parameters.
- This study proposes a Frequency Attention Convolution Module (FACM) that facilitates continuous extraction of low-level global spatial semantics. By alleviating feature misalignment at higher semantic levels, FACM significantly improves underwater target localization accuracy and reduces missed detections in turbid environments.
- This study introduces a Multi-dimensional Feature Enhancement Module (MFEM) designed to mitigate underwater noise and enhance multi-scale feature fusion through improved cross-dimensional semantic interactions. The proposed module significantly strengthens foreground perception robustness and effectively reduces false positives in high-turbidity environments.

The rest of this study is structured as follows. Section 2 introduces relevant deep learning algorithms for underwater object detection. In Section 3, the relevant theories of MFEM and FACM methods are introduced. The overall structure, which describes FSD-Net. Section 4 presents the experiments conducted with related methods on the datasets. Finally, Section 5 concludes the study.

## 2 Related studies

The emergence of deep learning has greatly improved the efficiency of object detection. Xu and Matzner (2018) applied the YOLO algorithm to train and detect underwater fish, achieving promising detection performance. Rosli et al. (2021) leveraged YOLOv4 to effectively detect marine animals in blurred and low-light underwater scenes. Merencilla et al. (2021) adopted YOLOv3 for accurate shark detection across various distances. However, the direct application of deep learning techniques in underwater environments is severely hindered by marine noise interference, preventing the models from reaching their full potential.

Image enhancement is an effective approach to mitigate noise in underwater images, thereby improving visual quality and feature clarity for subsequent detection tasks. Wei and Chen (2021) employed a generative adversarial network (GAN) to regenerate high-quality underwater images by suppressing noise conditions, thereby enhancing the effectiveness of model training. Peng et al. (2021) applied offline image enhancement to underwater training samples, effectively improving underwater target detection accuracy. Roy and Talukder (2024) employed channel-wise denoising using image filtering techniques, and integrated the results with YOLOv8, leading to enhanced accuracy in underwater object detection. Wang et al. (2022) refined the Retinex-based method to enhance underwater image quality and combined it with an optimized YOLO anchor box assignment strategy to further boost detection performance. Although underwater image enhancement can markedly improve model training and detection accuracy, it is a computationally intensive process that restricts real-time detection efficiency.

Enhancing spatial feature information effectively improves the localization ability of underwater targets. Noman et al. (2021) enhanced the feature extraction capability by modifying the backbone of Faster R-CNN, which led to improved detection performance for seagrass. Shi et al. (2021) proposed an enhanced high-level semantic fusion structure within Faster R-CNN to effectively improve the discrimination of underwater targets. Zhao et al. (2022) proposed a symmetric fusion attention mechanism to enhance deep semantic features and improve the integration of effective information. Li S. et al. (2022) improved underwater feature perception through weighted feature enhancement across multiple network stages. Hua et al. (2023) refined backbone features and incorporated a dynamic aggregation strategy to enhance underwater information fusion. However, solely enhancing high-level semantic features remains insufficient for precise feature alignment, often resulting in inaccurate localization of underwater targets.

In this study, we enhance deep semantic features while simultaneously extracting long-range dependencies from shallow spatial semantic information. This integration facilitates effective feature alignment and fusion across multiple scales and levels without introducing significant computational overhead, thereby mitigating missed and false detections in underwater object detection, as illustrated in Figure 2b.

## 3 Materials and methods

In this section, a network overview is provided in Section 3.1, following which the proposed Multi-dimensional feature enhancement module (MFEM) is described in Section 3.2. The proposed Frequency attention convolution module (FACM) is explained in Section s3.3.

### 3.1 FSD-Net

**Figure 4:**
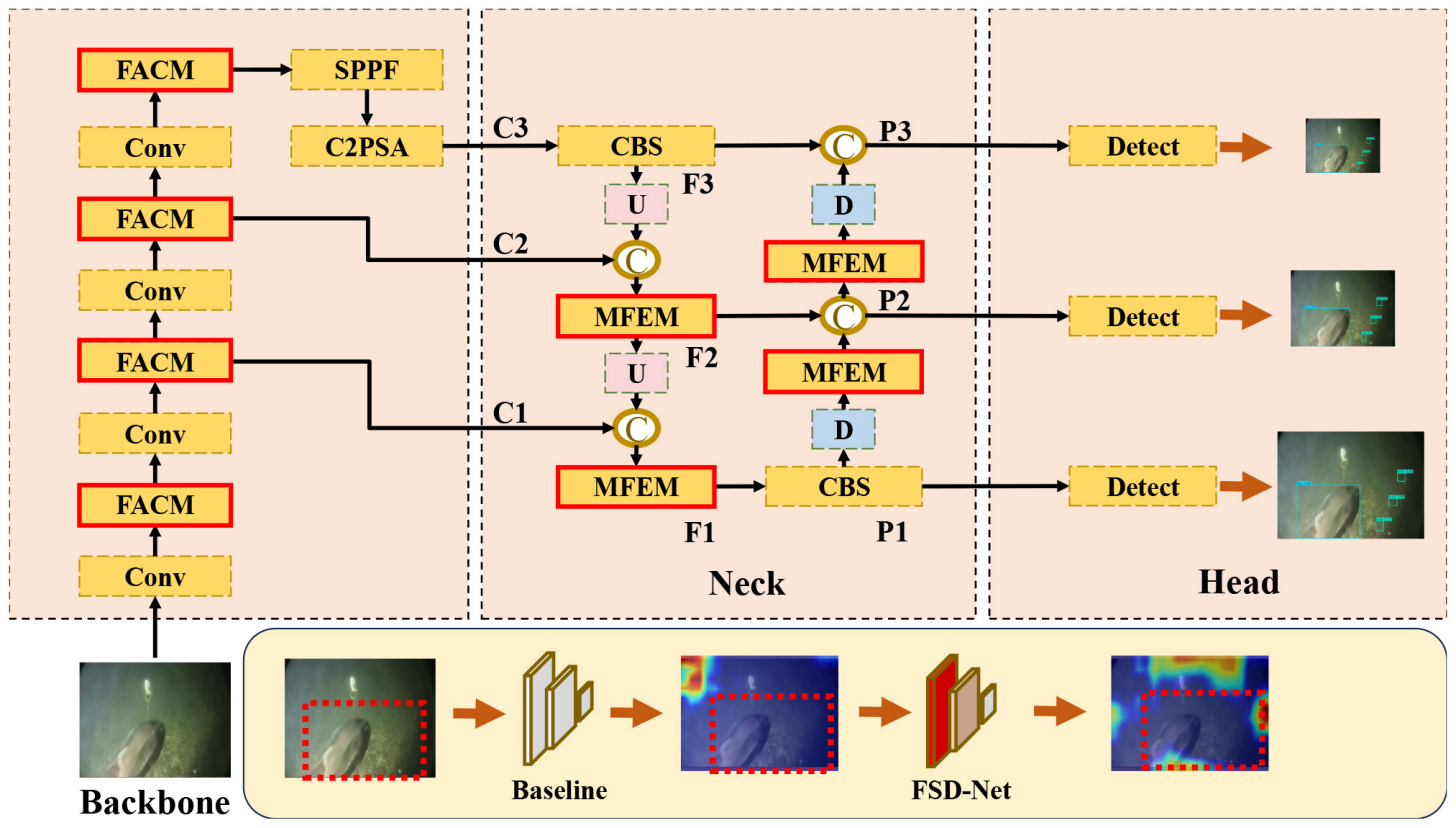
The structure of FSD-Net.

The overview of FSD-Net is presented in Figure 4. FSD-Net comprises three essential components: a backbone for feature extraction, a neck for feature fusion, and a head for prediction. The Frequency Attention Convolution Module (FACM) is integrated into the backbone and is responsible for effectively preserving and extracting low-level spatial semantic information, enabling better alignment between low-level and high-level features. The Multi-dimensional Feature Enhancement Module (MFEM), located in the neck, focuses on enhancing high-level semantic features to facilitate the fusion of informative cues. The explanation of the internal acronym structure of FSD-Net can be found in Section S5 of the Supplementary Section S5.

FSD-Net takes input images of dimensions *H*×*W*×3. The backbone network is exploited to extract multi-scale features via multi-layer convolutions, encompassing low-level edge and texture features, as well as high-level semantic features. The final three scale features are obtained through deep feature extraction by the backbone. The feature maps of the last three blocks can be defined as $C_{i}\in {\text{R}}^{h_{i}\times w_{i}\times c_{i}}\left(c_{i=\left\{1,2,3\right\}}=\left\{256,512,1024\right\}\right)$. And *h*_*i*_ and *w*_*i*_ can be formulated as *H*/2^*i*^ and *W*/2^*i*^. *C*_1_ represents the first-scale feature map obtained after the original image *I* is processed by the backbone for feature extraction. The process can be formulated in Equation (1):


$$C_{1}=FACM(Conv(FACM(Conv(Conv(I)))))$$ (1)

where *FACM* is the Frequency Attention Convolution Module, *Conv*(.) is a 3 × 3 convolution block. The process of obtaining *C*_2_ and *C*_3_ can be described in Equations (2) and (3):


$$\begin{array}{l} C_{2}=FACM\left(\left(Conv\left(C_{1}\right)\right)\right) \end{array}$$ (2)

$$C_{3}=C2PSA(SPPF(FACM((Conv(C_{2})))))$$ (3)

where *SPPF* is Spatial Pyramid Pooling Fast module, *C*2*PSA* is an attention mechanism. The features are then employed for further processing.

The neck structure plays a crucial role in integrating features from multi-level feature maps. Relying solely on single-level feature maps for prediction has proven inadequate, as it fails to effectively support the detection of objects at varying scales. To better leverage multi-level features, a pyramid structure is introduced to enhance feature fusion and reuse.

As shown in Figure 4, firstly, from the top to bottom, the neck network adopts a 3 × 3 convolution to process *C*_3_ and get *F*_3_, which can be formulated in Equation (4):


$$F_{3}=CBS(C_{3})$$ (4)

where *CBS*(.) is a Convolution with 3 × 3 kernel, Batch Normalization and SiLU function.

Then feature maps *C*_*i*_(*i* = 1, 2) and *F*_*i*_(*i* = 2, 3) are used as inputs, upsampling to align the channel and size of feature maps in different layers. Then adjacent feature maps are fused by a simple concatenation operation. The process can be defined in Equation (5):


$$F_{i}=MFEM(Concat(C_{i},Up(F_{i+1}))),\;i=1,2$$ (5)

where $F_{i}\in {\text{R}}^{h_{i}\times w_{i}\times c_{i}}\left(i=1,2,3\right)$, *MFEM*(.) is the method named Multi-dimensional feature enhancement module (MFEM), *Concat*(, ) is the concatenation operation, *Up*(.) is the 2 × upsampling operation by using bilinear interpolation. Secondly, from the bottom to top, the neck network adopts a 3 × 3 convolution to process *F*_1_ and get *p*_1_, which can be computed in Equation (6):


$$P_{1}=CBS(F_{1})$$ (6)

From the bottom to top, feature maps *F*_*i*_(*i* = 2, 3) and *P*_*i*_(*i* = 1, 2) are used as inputs, upsampling to align the channel and size of feature maps in different layers. Then adjacent feature maps are fused by a simple concatenation operation. The process can be defined in Equation (7):


$$P_{i}=MFEM(Concat(F_{i},Up(P_{i-1}))),\;i=2,3$$ (7)

where $P_{i}\in {\text{R}}^{h_{i}\times w_{i}\times c_{i}}\left(i=1,2,3\right)$. Finally, *P*_*i*_, as the features from the three scales, are employed for processing and output by the detection head.

### 3.2 Multi-dimensional feature enhancement module

Underwater noise can significantly interfere with the extraction of valid features, thereby hindering accurate object perception. Effective selection and enhancement of high-level semantic features can improve feature fusion quality and ultimately enhance the ability to perceive underwater targets. The specific characteristics of MFEM in underwater detection scenarios and its comparison with existing frequency-domain methods can be found in Supplementary Table S7.

**Figure 5:**
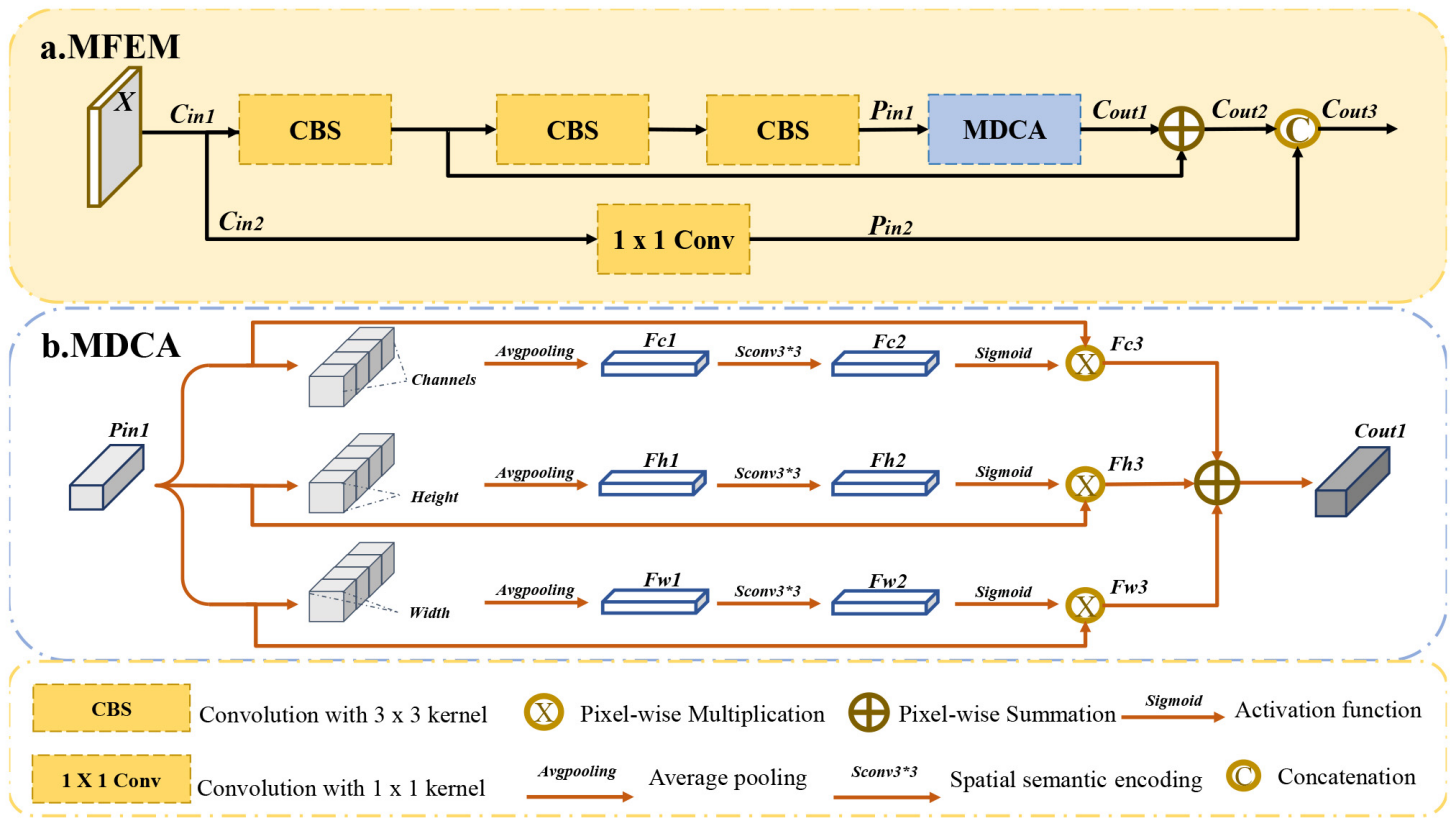
The structure of the Multi-dimensional feature enhancement module (MFEM).

The process is illustrated in Figure 5b. *P*_*in*1_ serves as the input for the three branches, with each branch performing weighting on the channel, height, and width dimensions of feature maps, respectively. First, each branch performs average pooling on *P*_*in*1_ to reduce the dimensions, resulting in *F*_*c*1_, *F*_*h*1_, and *F*_*W*1_. The process can be computed in Equation (8):


$$\begin{array}{l} F_{c1}=Avgpooling\left(permute\left(P_{in1}\right)\right)\\ F_{h1}=Avgpooling\left(permute\left(P_{in1}\right)\right)\\ F_{w1}=Avgpooling\left(permute\left(P_{in1}\right)\right) \end{array}$$ (8)

where $P_{in1}\in {\text{R}}^{h_{i}\times w_{i}\times c_{i}}$, $F_{c1}\in {\text{R}}^{1\times 1\times c_{i}}$, $F_{h1}\in {\text{R}}^{h_{i}\times 1\times 1}$ and $F_{w1}\in {\text{R}}^{1\times w_{i}\times 1}$, *Avgpooling* is an average pooling, *permute*(.) is a dimension rearrangement. The features with eliminated dimensions are then processed through dimension rearrangement and convolutional encoding. These processes can be described in Equation (9):


$$\begin{array}{l} F_{c2}=permute\left(Conv_{1*1}\left(permute\left(F_{c1}\right)\right)\right)\\ F_{h2}=permute\left(Conv_{1*1}\left(permute\left(F_{h1}\right)\right)\right)\\ F_{w2}=permute\left(Conv_{1*1}\left(permute\left(F_{w1}\right)\right)\right) \end{array}$$ (9)

where $F_{c2}\in {\text{R}}^{1\times 1\times c_{i}}$, $F_{h2}\in {\text{R}}^{h_{i}\times 1\times 1}$ and $F_{w2}\in {\text{R}}^{1\times w_{i}\times 1}$, *Conv*_1*1_ is a 1 × 1 convolution. Convolutional encoding is applied to integrate the semantic information of the corresponding dimensions, facilitating subsequent weighting. The weighting operation can be defined in Equation (10):


$$\begin{array}{l} F_{c3}=sigmoid\left(F_{c2}\right)⊗permute\left(P_{in1}\right)\\ F_{h3}=sigmoid\left(F_{h2}\right)⊗permute\left(P_{in1}\right)\\ F_{w3}=sigmoid\left(F_{w2}\right)⊗permute\left(P_{in1}\right) \end{array}$$ (10)

where *F*_*c*3_, *F*_*h*3_ and $F_{w3}\in {\text{R}}^{h_{i}\times w_{i}\times c_{i}}$. The weighted semantics from the three branches are fused by summation to get $C_{out1}\in {\text{R}}^{h_{i}\times w_{i}\times c_{i}}$.

The multi-dimensional collaborative attention (MDCA) is combined through residual concatenation and channel concatenation, resulting in the proposed Multi-dimensional Feature Enhancement Module (MFEM). As shown in Figure 5a, $X\in {\text{R}}^{h_{i}\times w_{i}\times c_{i}}$ is the input of the module, which can be divided into two branches. *C*_*in*1_ is processed to get *P*_*in*1_ by three *CBS* blocks. *P*_*in*1_ serves as the input of MDCA. The accuracy of feature extraction is ensured by combining residual addition and channel concatenation operations with the branch structure features. These processes can be computed in Equations (11) and (12):


$$\begin{array}{l} C_{out2}=MDCA\left(CBS\left(CBS\left(CBS\left(C_{in1}\right)\right)\right)\right)+CBS\left(C_{in1}\right) \end{array}$$ (11)

$$\begin{array}{l} C_{out3}=Concat\left(C_{out2},Conv_{1*1}\left(C_{in1}\right)\right) \end{array}$$ (12)

where $C_{out2},C_{out3}\in {\text{R}}^{h_{i}\times w_{i}\times c_{i}}$, *Conv*_1*1_(.) is a 1 × 1 convolution, *Concat*(.) is the concatenation operation. MFEM assigns adaptive weights across multiple feature dimensions to emphasize informative semantic content. This strategy effectively suppresses the influence of underwater noise and improves the robustness of feature representation. MFEM facilitates accurate classification of underwater targets by enhancing semantic feature representations across multiple dimensions. This enables the detector to make correct category predictions while maintaining precise localization, thereby effectively reducing false detections in underwater environments. The theoretical rationale and experimental validation for the selection of internal components in MFEM are provided in Supplementary Section S9.

### 3.3 Frequency attention convolution module

Low-level spatial features are essential for accurately localizing underwater objects. However, the misalignment between shallow spatial semantics and deep channel features may result in missed detections. Therefore, effectively extracting and preserving edge information of underwater targets is critical for enhancing detection accuracy in complex underwater environments.

**Figure 6:**
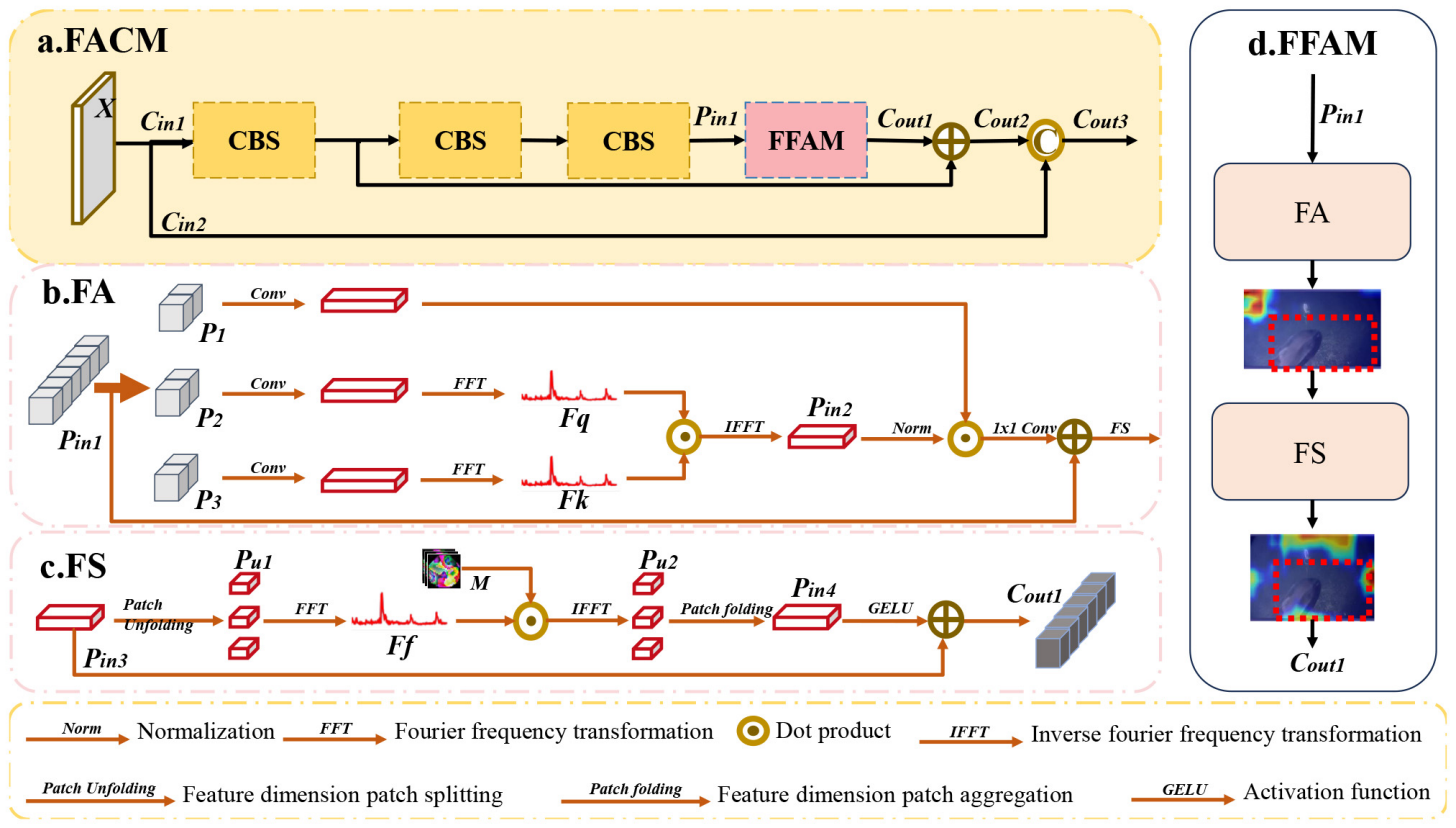
The structure of frequency attention convolution module (FACM).

As shown in Figures 6b, c, FACM is responsible for the transformation of spatial domain features into frequency domain signals and applies self-attention weighting. FACM includes Frequency Attention (FA) and Frequency strengthening (FS). The first stage of FFAM is generating frequency-domain attention weights via Query-Key interaction to filter noise-related frequency components. The second stage of FFAM uses a learnable vector to enhance target-related high-frequency features preserved by FA. The particularity of FACM in underwater detection scenarios and its comparative differences with existing frequency-domain methods can be found in Supplementary Table S6. *P*_*in*1_ serves as the input of FFAM. First, *P*_*in*1_ is split along the channel dimension to obtain $P_{i}\in R^{h_{i}\times w_{i}\times c_{i}/3},i=\{1,2,3\})$. *P*_2_ and *P*_3_ are transformed into frequency domain signals via the Fourier transform, serving as Query and Key for the dot-product to generate weights. The processes of transforming are defined in Equation (13):


$$\begin{array}{l} F_{q}=FFT\left(Conv\left(P_{2}\right)\right)\\ F_{k}=FFT\left(Conv\left(P_{3}\right)\right) \end{array}$$ (13)

where $F_{q},F_{k}\in {\text{R}}^{h_{i}\times w_{i}\times \left(\left(c_{i}/6\right)+1\right)}$, *FFT*(.) is Fourier transform, *Conv*(.) is a 3 × 3 convolution with normalization. Afterward, the inverse Fourier transform is applied to convert them back to spatial domain features, completing the weighting of the original value. The processes can be computed in Equations (14) and (15):


$$\begin{array}{l} P_{in2}=IFFT\left(F_{q}⊙F_{k}\right) \end{array}$$ (14)

$$P_{in3}=P_{in1}+Conv_{1*1}((Norm(P_{in2}⊙P_{1}))$$ (15)

where $P_{in2}\in {\text{R}}^{h_{i}\times w_{i}\times c_{i}/3},P_{in3}\in {\text{R}}^{h_{i}\times w_{i}\times c_{i}}$, *IFFT*(.) is Inverse fourier transform, *Norm* is normalization, *Conv*_1*1_ is a 1 × 1 convolution.

To ensure that the high-frequency information in the frequency domain signals corresponds to the feature semantics of the foreground targets, a learnable feature vector is introduced in Fs to form weights, ensuring the accuracy of the high-frequency information. As shown in Figure 6c, *P*_*in*3_ serves as the input of FS. *P*_*in*3_ is dimensionally sliced to ensure that the effective spatial domain semantic information is uniformly transformed into frequency domain signals. The process of transforming can be described in Equations (16) and (17):


$$\begin{array}{l} P_{u1}=Patch_{unfolding}\left(P_{in3}\right) \end{array}$$ (16)

$$\begin{array}{l} F_{f}=FFT\left(P_{u1}\right) \end{array}$$ (17)

where $P_{u1}\in {\text{R}}^{c_{i}\times h1_{i}\times w2_{i}\times h2_{i}\times w1_{i}}\left(h_{i}=h1_{i}\times h2_{i},w_{i}=w1_{i}\times w2_{i}\right)$, $F_{f}\in {\text{R}}^{c_{i}\times h1_{i}\times w2_{i}\times h2_{i}\times \left(\left(w1_{i}/2\right)+1\right)}$, *Patch*_*unfolding*_ is the operation of slicing. The transformed frequency domain signal *F*_*f*_ is multiplied by the learnable vector *M* for weighted attention, ensuring the accuracy of the high-frequency information. Afterward, *F*_*f*_is transformed back into spatial domain features. The feature dimensions are restored through aggregation, and the final fusion is achieved by a residual connection, resulting in *C*_*out*1_. The processes can be computed in Equations (18–20):


$$\begin{array}{l} P_{u2}=IFFT\left(M⊙F_{f}\right) \end{array}$$ (18)

$$\begin{array}{l} P_{in4}=Patch_{folding}\left(P_{u2}\right) \end{array}$$ (19)

$$\begin{array}{l} C_{out1}=GELU\left(P_{in4}\right)+P_{in3} \end{array}$$ (20)

where $P_{u2}\in {\text{R}}^{c_{i}\times h1_{i}\times w2_{i}\times h2_{i}\times w1_{i}}\left(h_{i}=h1_{i}\times h2_{i},w_{i}=w1_{i}\times w2_{i}\right)$, $P_{in4},C_{out1}\in {\text{R}}^{h_{i}\times w_{i}\times c_{i}}$, *Patch*_*folding*_ is the operation of aggregation for feature maps, *GELU*(.) is an activation function.

FACM is combined through residual concatenation and channel concatenation. As is shown in Figure 6a, $X\in {\text{R}}^{h_{i}\times w_{i}\times c_{i}}$ is the input of the module, which can be divided into two branches. *C*_*in*1_ is processed to get *P*_*in*1_ by three *CBS* blocks. *P*_*in*1_ serves as the input of FFAM. The accuracy of feature extraction is ensured by combining residual addition and channel concatenation operations with the branch structure features. These processes can be computed in Equations (21) and (22):


$$\begin{array}{l} C_{out2}=FFAM\left(CBS\left(CBS\left(CBS\left(C_{in1}\right)\right)\right)\right)+CBS\left(C_{in1}\right) \end{array}$$ (21)

$$\begin{array}{l} C_{out3}=Concat\left(C_{out2},C_{in2}\right) \end{array}$$ (22)

where $C_{out2},C_{out3}\in {\text{R}}^{h_{i}\times w_{i}\times c_{i}}$, *Concat*(.) is the concatenation operation.

FACM extracts and preserves global spatial semantic information by leveraging transformations between the spatial and frequency domains, thereby facilitating better alignment of multi-level feature semantics. The theoretical basis for how frequency-domain transformations in FACM facilitate the fusion of spatial semantic information and high-level semantic information can be found in Supplementary Section S8. The integration of enriched spatial information enhances the model's capacity to perceive the positional attributes of underwater targets, effectively reducing missed detections.

## 4 Experimental results

### 4.1 Datasets and evaluation metrics

UTDAC2020 (Liang and Song, 2022): Underwater dataset from the 2020 Underwater Target Detection Algorithm Competition. It contains 5,168 training and 1,293 validation images across four classes: echinus, holothurian, starfish, and scallop. Images available in four resolutions: 3840 × 2160, 1920 × 1080, 720 × 405, and 586 × 480.

Brackish dataset (Pedersen et al., 2019): Features annotated sequences of fish, crabs, and starfish captured in Brackish water under varying visibility conditions, representing the initial phase of a long-term marine monitoring project. All datasets are randomly split into 80% training and 20% test sets.

To comprehensively evaluate performance, this study employs average precision (AP) as the primary metric. Specifically, AP@0.5 and AP@0.75 denote the average precision at thresholds of 0.5 and 0.75, respectively. Mean average precision (mAP), calculated as the average AP at a threshold of 0.5, serves as the overall evaluation metric. Additionally, Recall, Precision, and F1-score are utilized to further assess model performance.

### 4.2 Implementation details

All experiments are conducted on an NVIDIA GeForce RTX 2080 SUPER GPU. Network parameters are initialized with a normal distribution (Zhao et al., 2024). We utilize the AdamW optimizer (Zhou et al., 2024) with a weight decay of 0.0001 and momentum of 0.9. Training uses a batch size of 4 and an initial learning rate of 0.01, which is reduced by a factor of 0.1 at epochs 24 and 30 during a total of 50 epochs. Input images are uniformly resized to 640 × 640 pixels for both training and inference. Detailed hyperparameters, training configurations, and image preprocessing strategies of FSD-Net and the compared models are provided in the Supplementary Sections S1, S3.

### 4.3 Experiments on UTDAC2020 dataset

#### 4.3.1 Ablation experiments on UTDAC2020 dataset

To validate the effectiveness of the proposed architecture, a series of ablation experiments is conducted, with the results presented in Table 1. Following FACM's enhancement of global spatial semantic feature extraction via frequency domain transformation, the model achieves improvements of 1.7% in *AP*_50_ and 2.7% in *AP*_75_. Within the fusion structure, MFEM improves the baseline through multi-dimensional feature enhancement, leading to a 1.2% increase in *AP*_50_ and a 0.9% gain in *AP*_75_. When FACM and MFEM are combined, the baseline model attains a 3.8% increase in *AP*_50_ and a 3.6% improvement in *AP*_75_. To further verify the effectiveness and complementary advantages of the proposed FACM and MFEM modules, we conduct comprehensive ablation experiments on the UTDAC2020 dataset with the additional metric mAP@[0.5:0.95]. As shown in Table 1, the baseline model without any modules achieves 42.8% mAP@[0.5:0.95]. When only FACM is integrated, the mAP@[0.5:0.95] is improved to 44.9%, demonstrating that FACM effectively enhances feature alignment and preserves low-level spatial information. When only MFEM is applied, the performance is increased to 43.5% mAP@[0.5:0.95], validating that MFEM suppresses background noise and enhances discriminative features. By combining both FACM and MFEM, FSD-Net reaches 48.7% mAP@[0.5:0.95], which significantly outperforms the baseline and all individual module settings. These results confirm the effectiveness and synergies of the two proposed modules, and fully validate the rationality of the overall FSD-Net framework.

**Table 1:** Ablation analysis on UTDAC2020 dataset.

FACM	MFEM	AP_50_	AP_75_	mAP@[0.5:0.95]	Params	FLOPs
✗	✗	81.9	47.3	42.8	9.4 M	21.3 G
✓	✗	83.6	49.7	44.9	9.5 M	21.4 G
✗	✓	82.1	48.2	43.5	9.7 M	22.6 G
✓	✓	**85.7**	**50.9**	**48.7**	9.8 M	22.8 G

The best performance metrics are highlighted in bold.

To further validate the effectiveness of FACM and MFEM, we conducted additional ablation experiments from multiple perspectives. These experiments include the impact of different module embedding positions, the effects of varying kernel sizes in frequency-domain processing, and comparisons across different types of attention mechanisms. Detailed experimental results and analyses can be found in Supplementary Table S10, Section S11. Visualization of the semantic effects of MFEM and FACM on underwater targets is provided in Supplementary Section S4.

#### 4.3.2 Performance analysis of FSD-Net

As shown in Table 2, this study compares the performance of FSD-Net with current state-of-the-art algorithms on the UTDAC2020 dataset. Among the one-stage lightweight algorithms, YOLOv13s achieves an F1-score of 80.4%, while the large-scale model YOLOv9L reaches 81.8%. Among the latest DETR-based methods, Define-s achieves an F1-score of 82.0%. FSD-Net achieves an F1-score of 82.5%, outperforming other state-of-the-art methods. More metrics can be seen in Table 3 FSD-Net achieves an AP50 of 85.7%, which is 2.1% higher than the next-leading method De-fine-s (83.1%) and 3.8 pp higher than the strong baseline YOLOv13s (82.5%). This significant performance advantage confirms that FSD-Net possesses superior capability in accurately detecting underwater targets even under the challenging conditions of the UTDAC2020 dataset.

**Table 2:** Comparison of object detection methods for more metrics.

Method	Recall	Precision	F1-score
Faster R-CNN (Ren et al., 2016)	78.3	85.2	81.6
YOLOv5s (Wu et al., 2022)	78.2	81.5	79.8
YOLOv6s (Li C. et al., 2022)	77.2	76.0	76.6
YOLOv8s (Terven et al., 2023)	78.3	80.7	79.5
YOLOv9L (Wang C.-Y. et al., 2024)	79.5	84.2	81.8
YOLOv10s (Wang A. et al., 2024)	79.2	80.6	79.9
YOLOv12s (Tian et al., 2025)	80.2	81.3	80.7
YOLOv13s (Lei et al., 2025)	78.9	82.1	80.4
Boosting R-CNN (Song et al., 2023)	**81.2**	80.3	80.7
LightWeight (Yeh et al., 2021)	72.5	76.7	74.5
APAN (Yu et al., 2023)	73.2	71.3	72.2
FMSPP (Hua et al., 2023)	78.3	82.6	80.4
UW-YOLOv8 (Guo et al., 2024)	77.7	84.3	80.9
Define-s (Gao M. et al., 2024)	79.2	85.1	82.0
DEIM-s (Huang et al., 2025)	78.6	84.2	81.3
**FSD-Net**	79.1	**86.1**	**82.5**

The best performance metrics are highlighted in bold.

**Table 3:** Performance comparison on UTDAC2020 dataset (including mAP@[0.5:0.95]).

Method	*AP*_50_	*AP*_75_	mAP@[0.5:0.95]
Faster R-CNN (Ren et al., 2016)	80.9	44.1	41.2
YOLOv5s (Wu et al., 2022)	80.3	47.3	42.5
YOLOv6s (Li C. et al., 2022)	76.7	43.7	39.8
YOLOv8s (Terven et al., 2023)	79.6	45.6	43.1
YOLOv9L (Wang C.-Y. et al., 2024)	83.8	52.8	47.6
YOLOv10s (Wang A. et al., 2024)	78.5	46.3	42.9
YOLOv12s (Tian et al., 2025)	81.9	48.9	44.3
YOLOv13s (Lei et al., 2025)	82.5	48.6	45.2
Boosting R-CNN (Song et al., 2023)	82.4	52.5	46.8
LightWeight (Yeh et al., 2021)	76.4	44.2	38.9
APAN (Yu et al., 2023)	79.6	45.7	41.5
FMSPP (Hua et al., 2023)	83.6	**53.8**	47.3
UW-YOLOv8 (Guo et al., 2024)	83.6	50.3	46.1
Define-s (Gao M. et al., 2024)	83.1	49.2	46.5
DEIM-s (Huang et al., 2025)	82.8	48.7	45.9
**FSD-Net**	**85.7**	**53.8**	**48.7**

The best performance metrics are highlighted in bold.

**Figure 7:**
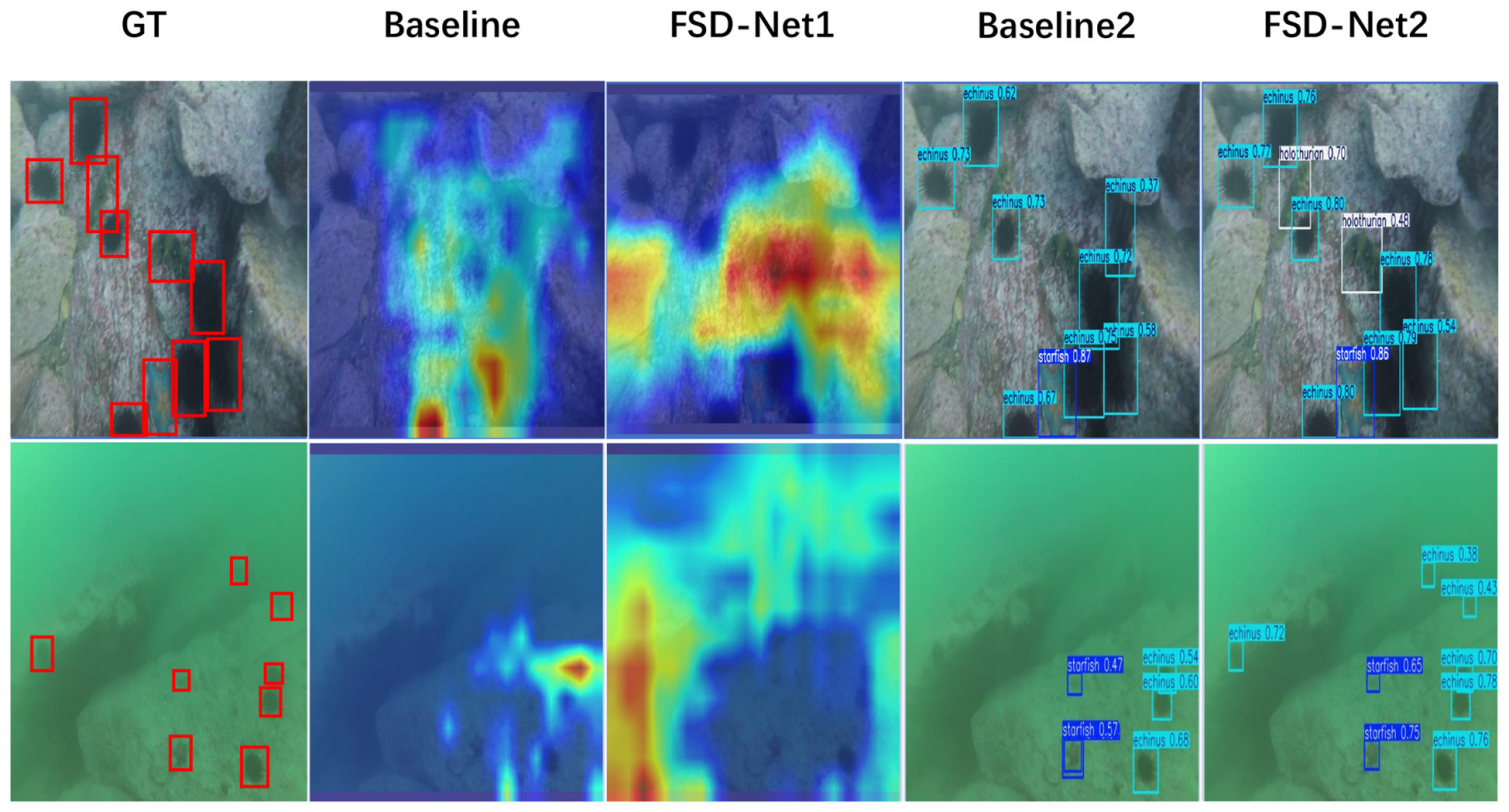
Comparison of heatmap perception and detection performance between FSD-Net and baseline models on the UTDAC2020 dataset.

**Figure 8:**
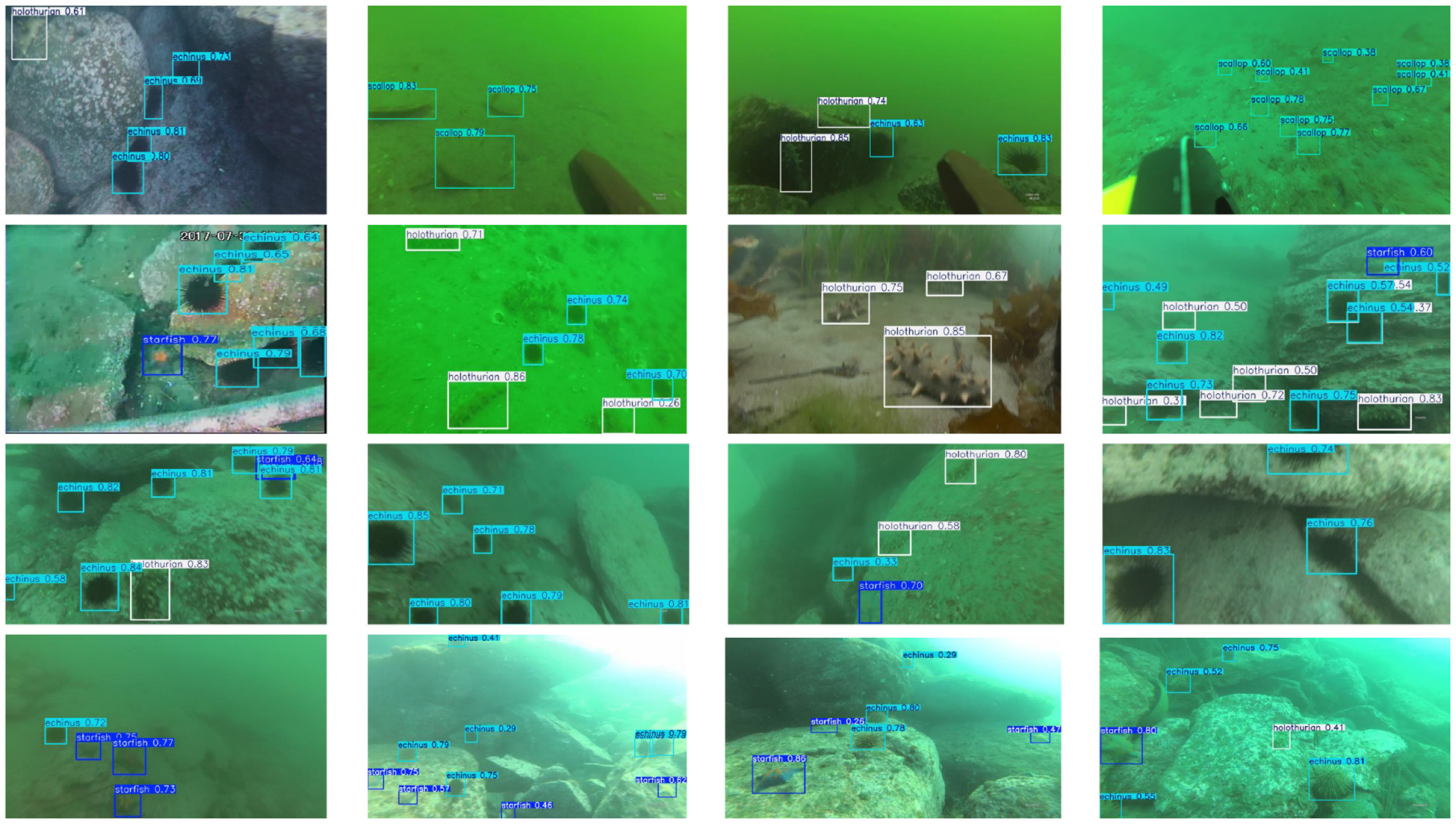
The detection performance of FSD-Net on the UTDAC2020 dataset.

As shown in Figure 7, we compare the performance of FSD-Net and the baseline using heatmap-based visualization. In the column rendered by FSD-Net, underwater targets exhibit higher intensity responses, indicating that our method has superior perception capabilities for underwater objects compared to the baseline. The comparison in the detection results column further demonstrates that FSD-Net effectively alleviates the missed detection problem. More comprehensive real-world detection results are presented in Figure 8, where FSD-Net accurately detects underwater targets of various sizes and types.

To conduct a detailed analysis of FSD-Net's detection capabilities for dense targets, low-visibility scenarios, and small targets in underwater scenes, experiments were performed on the UTDAC2020 and RUOD datasets. Performance metrics and visualized detection results can be found in Supplementary Tables S17, S18, and Supplementary Figures S2, S3. The experimental results further demonstrate the effectiveness and generalization of FSD-Net in underwater detection tasks.

### 4.4 Experiments on Brackish dataset

#### 4.4.1 Ablation experiments on Brackish dataset

To verify the effectiveness of our method in different underwater environments, we conduct experiments on the Brackish dataset. Table 4 presents the ablation study of FACM and MFEM on the baseline. After FACM improves the global spatial semantic feature extraction through frequency domain transformation, an improvement of 2.4% in *AP*_50_ and 1.3% in *AP*_75_ is achieved. In the model fusion structure, MFEM enhances the baseline model by multi-dimensional feature enhancement, resulting in a 2.9% increase in *AP*_50_ and a 1.8% improvement in *AP*_75_. Under the combined effect of FACM and MFEM, the baseline model achieves a 3.9% increase in *AP*_50_ and a 1.6% improvement in *AP*_75_. To verify the generalization ability of FACM and MFEM, we further conduct ablation experiments on the Brackish dataset with the additional metric mAP@[0.5:0.95]. As illustrated in Table 4, the baseline model yields 83.2% mAP@[0.5:0.95]. When introducing FACM alone, mAP@[0.5:0.95] is improved to 85.6%. When employing MFEM alone, the performance is enhanced to 86.2% mAP@[0.5:0.95]. By integrating both modules, FSD-Net achieves 88.9% mAP@[0.5:0.95], which consistently surpasses all other settings. The consistent performance improvements on both datasets demonstrate that FACM and MFEM work collaboratively to boost detection accuracy, localization precision, and comprehensive performance, further verifying the robustness and generalization of the proposed modules.

**Table 4:** Ablation analysis on Brackish dataset.

FACM	MFEM	AP_50_	AP_75_	mAP@[0.5:0.95]	Params	FLOPs
✗	✗	94.2	78.4	83.2	9.4 M	21.3 G
✓	✗	96.6	79.7	85.6	9.5 M	21.4 G
✗	✓	97.1	80.2	86.2	9.7 M	22.6 G
✓	✓	**98.1**	**82.0**	**88.9**	9.8 M	22.8 G

The best performance metrics are highlighted in bold.

Further ablation analyses can be found in Supplementary Table S10, Supplementary Section S11.

#### 4.4.2 Performance analysis of FSD-Net

Table 5 presents the performance comparison of our method on the Brackish dataset. Among the one-stage lightweight algorithms, YOLOv12s achieves an F1-score of 95.4%, while YOLOv13s reaches 95.3%. Among the latest DETR-based methods, Define-s achieves an F1-score of 95.0%. FSD-Net achieves the highest performance with an F1-score of 97.0%, outperforming all other state-of-the-art methods. As shown in Table 6, FSD-Net achieves an exceptional AP50 of 98.1%, which is 2.9% higher than the next-leading method, YOLOv13s (95.4%), and 2.7 pp higher than De-fine-s (95.2%). This near-perfect detection accuracy underscores FSD-Net's strong robustness in underwater target detection under the scenario of the Brackish dataset.

**Table 5:** Comparison of object detection methods on Brackish.

Method	Recall	Precision	F1-score
Faster R-CNN (Ren et al., 2016)	93.4	92.5	92.9
YOLOv5s (Wu et al., 2022)	91.4	90.3	90.8
YOLOv6s (Li C. et al., 2022)	89.5	88.3	88.9
YOLOv8s (Terven et al., 2023)	89.2	90.5	89.8
YOLOv9L (Wang C.-Y. et al., 2024)	94.4	91.8	93.1
YOLOv10s (Wang A. et al., 2024)	93.7	90.2	91.9
YOLOv12s (Tian et al., 2025)	96.2	94.6	95.4
YOLOv13s (Lei et al., 2025)	95.3	95.4	95.3
Boosting R-CNN (Song et al., 2023)	94.9	91.5	93.2
LightWeight (Yeh et al., 2021)	90.5	88.2	89.3
APAN (Yu et al., 2023)	91.3	89.2	90.2
FMSPP (Hua et al., 2023)	92.1	90.2	91.1
UW-YOLOv8 (Guo et al., 2024)	90.3	91.2	90.7
Define-s (Gao M. et al., 2024)	94.8	95.2	95.0
DEIM-s (Huang et al., 2025)	97.2	94.3	95.7
**FSD-Net**	**97.5**	**96.5**	**97.0**

The best performance metrics are highlighted in bold.

**Table 6:** Performance comparison on Brackish dataset (including mAP@[0.5:0.95]).

Method	*AP*_50_	*AP*_75_	mAP@[0.5:0.95]
Faster R-CNN (Ren et al., 2016)	90.2	75.3	78.6
YOLOv5s (Wu et al., 2022)	83.1	62.5	71.3
YOLOv6s (Li C. et al., 2022)	88.3	69.2	76.5
YOLOv8s (Terven et al., 2023)	88.7	70.2	77.1
YOLOv9L (Wang C.-Y. et al., 2024)	89.9	70.3	79.4
YOLOv10s (Wang A. et al., 2024)	88.2	70.1	78.9
YOLOv12s (Tian et al., 2025)	94.6	83.5	85.4
YOLOv13s (Lei et al., 2025)	95.4	**84.2**	86.1
Boosting R-CNN (Song et al., 2023)	91.5	77.3	82.8
LightWeight (Yeh et al., 2021)	87.4	69.3	76.8
APAN (Yu et al., 2023)	88.6	69.5	77.5
FMSPP (Hua et al., 2023)	90.3	74.8	81.2
UW-YOLOv8 (Guo et al., 2024)	89.6	72.3	80.5
Define-s (Gao M. et al., 2024)	95.2	83.8	87.0
DEIM-s (Huang et al., 2025)	94.3	82.9	86.4
**FSD-Net**	**98.1**	84.0	**88.9**

The best performance metrics are highlighted in bold.

**Figure 9:**
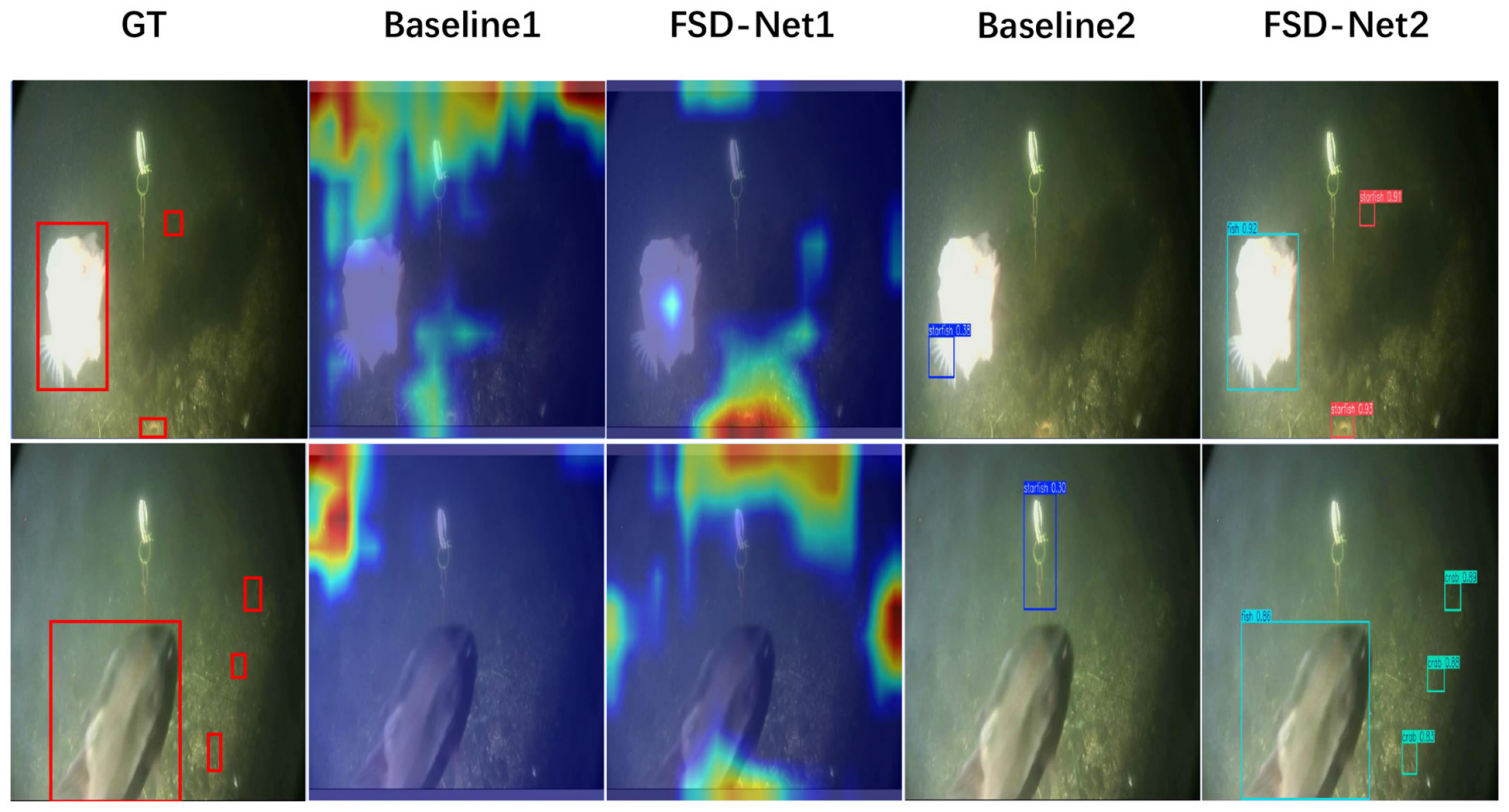
Comparison of heatmap perception and detection performance between FSD-Net and baseline models on the Brackish dataset.

**Figure 10:**
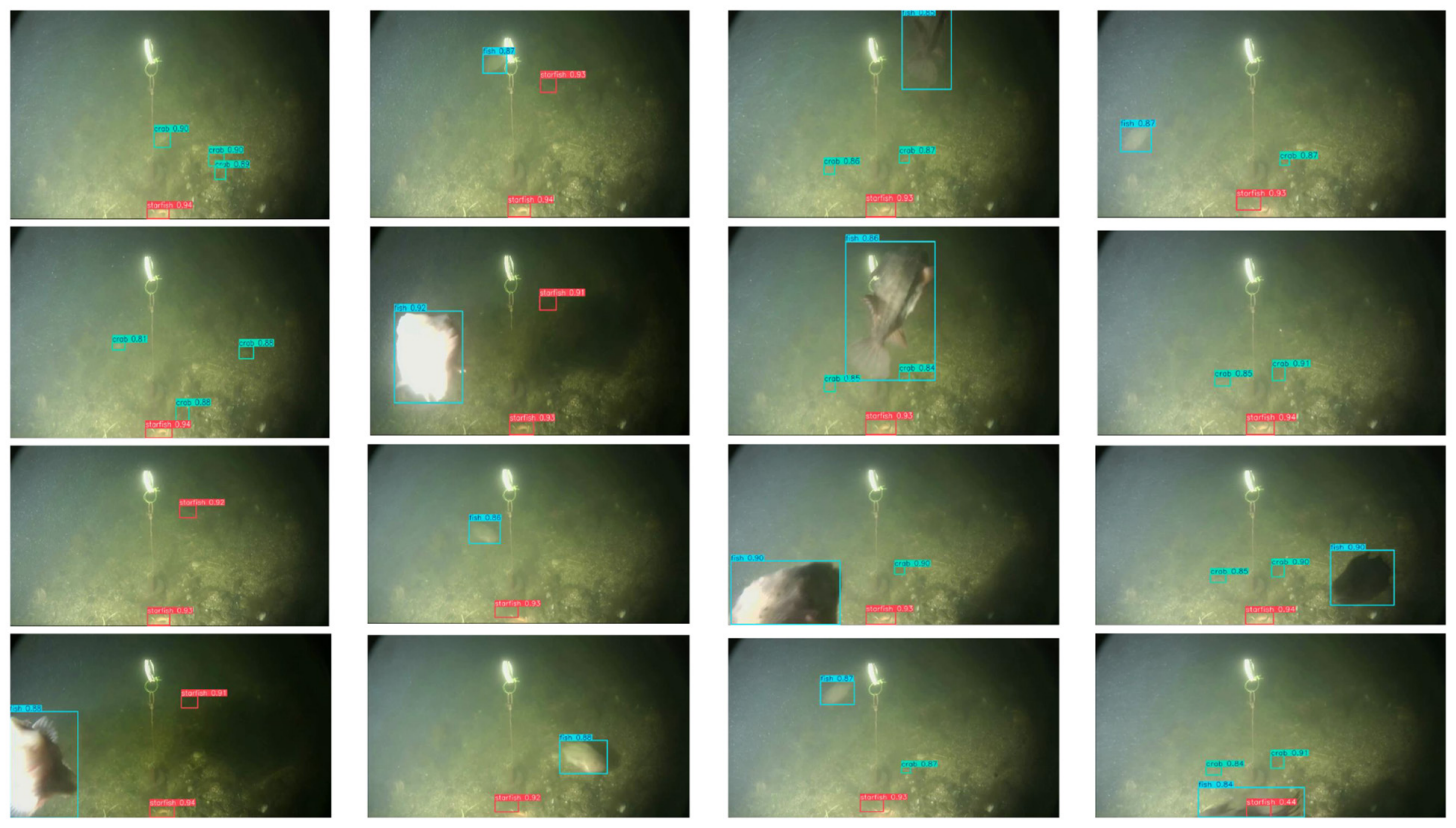
The detection performance of FSD-Net on the Brackish dataset.

As shown in Figure 9, we compare the performance of FSD-Net and the baseline through heatmap-based visualization. In the FSD-Net heatmap column, our method demonstrates enhanced spatial localization and more accurate rendering of targets compared to the baseline. The comparison in the detection results column shows that FSD-Net can effectively mitigate the false detection problem. More comprehensive real-world detection results are illustrated in Figure 10. In saline water environments with severe noise interference, FSD-Net is capable of accurately detecting underwater targets of various sizes and types.

Due to the relatively homogeneous scenarios in the Brackish dataset, we additionally utilized the RUOD dataset and conducted experiments on both the UTDAC2020 and RUOD datasets to analyze FSD-Net's detection capabilities for dense targets, low-visibility scenarios, and small targets in underwater scenes. Performance metrics and visualized detection results can be found in Supplementary Tables S17, S18, Figures S2, S3. The experimental results further demonstrate the generalization ability of FSD-Net across different types of underwater targets.

The variation in the performance ranking of different detectors across the UTDAC2020 and Brackish datasets is attributed to the inherent characteristic differences of the two datasets and the task adaptation bias of traditional model design. A detailed analysis of the causes of this phenomenon and the cross-dataset adaptability mechanism of FSD-Net is presented in Supplementary Section S13. The core advantage of FSD-Net is that it abandons the single-scenario optimization design of traditional models and designs the FACM and MFEM modules for the universal core challenges of underwater detection (noise interference, feature misalignment, and multi-scale target imbalance), thus realizing stable and superior detection performance on both turbid and clear underwater datasets.

### 4.5 Limitation and discussion

As shown in Supplementary Table S8, the accuracy and real-time performance are further analyzed. FACM and MFEM enhance the perception of underwater targets by extracting long-range spatial semantic features and performing multi-dimensional feature enhancement. However, compared with standard convolution operations, dimensional transformations and multi-dimensional feature enhancement introduce deeper network structures and higher computational complexity. The real-time performance analysis of the frequency-domain method FACM and the attention mechanism MFEM is presented in the Supplementary Section S2, Supplementary Tables S1–S3.

FSD-Net's competitive computational efficiency stems from the lightweight plug-and-play design of the FACM and MFEM modules, paired with task-specific efficient operations tailored for underwater detection, rendering its 9.8 M parameters and 22.8 GFLOPs highly rational. Specifically, FACM replaces O(N^2^) dense convolution with O(N log N) FFT/IFFT for global spatial-semantic alignment, effectively curbing GFLOPs growth while suppressing underwater scattering noise. MFEM employs a sparse multi-dimensional weighting mechanism to avoid redundant computation on background and noise features, thus preventing unnecessary parameter expansion. Both modules are embedded into the YOLOv11s baseline without introducing extra convolutional stacks or fully connected layers. As shown in Table 7, FSD-Net strikes a favorable accuracy-efficiency trade-off: it achieves 22.8 GFLOPs (the lowest among lightweight baselines) and 9.8 M parameters (fewer than YOLOv8s), while delivering superior detection performance across all underwater datasets. Despite a slightly lower FPS of 49.0 compared to the fastest lightweight detectors, FSD-Net maintains a competitive inference speed that satisfies the requirements of most practical underwater detection scenarios, fully validating the rationality of its computational design.

**Table 7:** Comparison of complexity and real-time performance.

Method	*GFLOPs*	*Params*(*M*)	*FPS*
Faster RCNN	282.8	60.2	16
YOLOv5s	23.8	9.1	53.2
YOLOv8s	28.4	11.1	55.6
YOLOv10s	24.8	**8.1**	**60.8**
FSD-Net	**22.8**	9.8	49.0

The best performance metrics are highlighted in bold.

However, compared with the original standard convolution operations of the YOLOv11s baseline, the dimensional transformations of FACM and multi-dimensional feature enhancement of MFEM introduce a small amount of computational overhead, resulting in a slight increase in FLOPs, 22.8 GFLOPs compared with the baseline 21.3 GFLOPs. Achieving an efficient and lightweight design, while balancing detection speed and accuracy, will be the primary focus of our future studies. A detailed analysis of the limitations and corresponding improvement plans can be found in Supplementary Section S12.

## 5 Conclusion

In this study, we propose a novel end-to-end underwater detection paradigm with inherent feature-domain enhancement, which unifies adaptive noise suppression and spatial-semantic alignment into the feature extraction process, rather than using independent image enhancement or simple module stacking. Specifically, it introduces two feature enhancement modules: the Frequency Attention Convolution Module (FACM), which aims to preserve global spatial semantic information through frequency domain transformation, enabling better alignment with deep channel semantic information and improving the localization of underwater objects; and the Multi-dimensional Feature Enhancement Module (MFEM), which strengthens information interactions across different dimensions in high-level features, performs secondary feature enhancement, and improves the fusion of underwater semantic features. Extensive experiments conducted on the UTDAC2020 and Brackish underwater datasets demonstrate that FSD-Net outperforms other state-of-the-art methods in underwater object recognition.

## Data Availability

The original contributions presented in the study are included in the article/Supplementary material, further inquiries can be directed to the corresponding author.
