# Supplementary material for: FSD-Net: underwater object detection based on frequency and spatial domain feature enhancement
Source: Front Artif Intell. 2026 Apr 17;9:1770342. doi: 10.3389/frai.2026.1770342 (PMC13133022; doi:10.3389/frai.2026.1770342)
Supplement: Supplementary file 1 [file Data_Sheet_1.pdf]

Supplementary materials

Paper title: FSD-Net:underwater object detection based on frequency and spatial domain feature enhancement

S1. Details of model parameter settings

To ensure the fairness and reproducibility of the performance comparison, the YOLOv11s baseline adopts the official open-source configuration (https://github.com/ultralytics/ultralytics) with no additional modifications, and all training hyperparameters, data processing strategies, and hardware environments are strictly consistent with those of the proposed FSD-Net. The detailed training recipe is as follows:

1. Model Configuration: The YOLOv11s baseline uses the default network structure of YOLOv11s, including the backbone , neck and head. The anchor settings are the official default values for small-scale models: three anchor boxes per scale, with sizes (10,13), (16,30), (33,23) for the small scale, (30,61), (62,45), (59,119) for the medium scale, and (116,90), (156,198), (373,326) for the large scale. The loss function follows the official YOLOv11 design, consisting of classification loss (BCEWithLogitsLoss), regression loss (CIoULoss), and objectness loss (BCEWithLogitsLoss), with weight coefficients of 1.0, 5.0, and 1.0, respectively.

2. Training Hyperparameters: The optimizer adopts AdamW, with a weight decay of 0.0001, betas=(0.9, 0.999), and eps=1e-8. The initial learning rate is set to 0.01, and a cosine annealing learning rate schedule is used, with a warm-up period of 5 epochs (warm-up learning rate from 0.001 to 0.01) to avoid unstable training in the early stage. The batch size is 4 (consistent with FSD-Net) due to hardware memory constraints, and the total training epochs are 50. The input resolution of all training and test images is 640×640, with letterbox padding used to maintain the aspect ratio of the original images, avoiding feature distortion.

3. Data Preprocessing and Augmentation: The data preprocessing steps for the YOLOv11s baseline are identical to those of FSD-Net: (1) Image normalization: Each pixel value is divided by 255 to scale to the range [0,1]; (2) Label processing: Bounding box coordinates are converted from absolute coordinates to relative coordinates (relative to the image width and height), and class labels are one-hot encoded. The data augmentation strategies are also consistent, including random cropping (crop scale range 0.5–1.0), horizontal flipping (probability p=0.5), brightness/contrast adjustment (±0.2), Gaussian blur (kernel size=3, probability p=0.3), and underwater scattering simulation (probability p=0.2, simulating the light scattering effect in underwater environments to enhance the generalization ability of the model). No additional augmentation strategies are used to avoid artificial optimization of the baseline.

4. Training Details: The training process uses gradient clipping with a maximum norm of 1.0 to prevent gradient explosion. Early stopping is adopted, with a patience of 10 epochs—if the validation mAP@[0.5:0.95] does not improve for 10 consecutive epochs, the training is stopped early, and the model with the highest validation mAP is saved as the final baseline model. The training is conducted on a single NVIDIA GeForce RTX 2080 SUPER GPU, with Intel Core i7-10700K CPU and 32GB RAM, ensuring consistent hardware environment with FSD-Net.

5. Evaluation Settings: The evaluation metrics for the YOLOv11s baseline are the same as those of FSD-Net, including AP50, AP75, mAP@[0.5:0.95], Recall, F1-scores and Precision. The evaluation is conducted on the test set of the UTDAC2020 dataset, with no data leakage between the training set, validation set, and test set (training set: 70%, validation set: 15%, test set: 15%).

S2. Comprehensive Runtime Analysis of FACM: Focus on FFT/IFFT, Patch Operations and GPU Kernel Performance

The experimental deployment was conducted on a hardware platform consisting of an NVIDIA GeForce RTX 2080 SUPER (8GB VRAM), Intel Core i9-10900K CPU (3.7GHz), and 32GB DDR4 RAM (3200MHz), with a software environment of PyTorch 2.1.0, CUDA 11.8, CuDNN 8.7.0, and Ubuntu 20.04 LTS. The test configuration adhered to real-world underwater robot deployment scenarios: input image resolution was fixed at 640×640 , batch size = 1 (to simulate sequential inference), 100 warm-up runs were performed to eliminate GPU initialization and kernel loading overhead, and 1000 test runs were conducted to calculate stable average latency. Latency was measured using torch.cuda.synchronize before and after each inference step to avoid asynchronous execution bias, with three key components quantified separately: end-to-end latency of complete networks, standalone latency of proposed modules (FACM and MFEM), and latency of the baseline YOLOv11s for direct comparison.

Table S1. Runtime Breakdown of Key Operations in FACM

Operation Type	Average Latency per Run (ms)	Proportion of Total FACM Latency (%)	Contribution to Total FLOPs of FACM (%)
Fast Fourier Transform (FFT)	0.82	18.2	18.2
Inverse FFT (IFFT)	0.78	17.5	17.5
Patch Unfolding/Folding	0.35	8.3	8.3
Frequency-domain Attention Weighting	0.35	56.0	56.0

The detailed breakdown of latency for each core operation in FACM is presented in Table S11. It should be noted that FFT/IFFT operations, despite their relatively low FLOPs contribution, account for a significant portion of the actual latency due to GPU kernel launch and data transformation overhead. In contrast, patch unfolding/folding and attention weighting operations have lower latency but are still indispensable for preserving spatial information and enhancing feature discrimination.

To further verify the efficiency of the proposed frequency-domain module in the entire network, we compared the end-to-end inference latency of FSD-Net with state-of-the-art underwater detectors under the same hardware/software configuration. As shown in Table S12, the total inference latency of FSD-Net is 20.4 ms (FPS = 49.0), where FACM only contributes 11.3% (2.3 ms) to the total latency. Compared with other detectors with similar accuracy (e.g., Define-s), FSD-Net achieves a 14.1% reduction in latency, demonstrating the efficiency of our frequency-domain module design.

Table S2. End-to-End Inference Latency Comparison (Batch Size = 1)

Method	Backbone	Total Inference Latency (ms)	FPS	Latency Contribution of Frequency-Domain/Attention Modules (ms)
YOLOv8s	C2f+Darknet50	18.0	55.6	-
YOLOv10s	Darknet50	17.5	60.8	-
Define-s	-	23.7	42.2	-
YOLOv11s	Darknet50	17.8	56.1	-
YOLOv11s+FACM	-	21.3	50.1	2.30

To further verify the efficiency of the proposed frequency-domain module in the entire network, we compared the end-to-end inference latency of FSD-Net with state-of-the-art underwater detectors under the same hardware/software configuration. As shown in Table S12, the total inference latency of YOLOv11s+FACM is 21.3 ms (FPS = 50.1), where FACM only contributes 2.3 ms to the total latency.

The runtime analysis confirms that the proposed FACM module achieves a good balance between feature enhancement and computational efficiency. The key reasons are as follows: (1) The block-wise FFT strategy reduces the computational overhead of global frequency-domain transformation, making it more GPU-friendly compared to full-image FFT (e.g., FFNet); (2) The integration of frequency-domain attention weighting with residual connections avoids redundant computations, ensuring that the latency increase is negligible relative to the performance gain; (3) In practical underwater robot deployment scenarios (where batch size

is typically 1), the 20.4 ms latency of FSD-Net meets the real-time requirement (≥30 FPS), providing a feasible solution for autonomous underwater exploration missions.

To clarify the latency contribution of each proposed module, we first analyzed the standalone runtime of FACM and MFEM within the complete network. The results are presented in Table S3, showing that both modules introduce moderate latency while providing significant performance gains.

Table S3. Runtime Breakdown of Proposed Modules (FACM + MFEM)

Module	Core Operations	Average Latency per Run (ms)	Proportion of Total FSD-Net Latency	Contribution to Total FLOPs of FSD-Net
FACM	Block-wise FFT/IFFT, Patch Unfolding/Folding, Frequency-domain Attention Weighting	2.30	10.8	49.1
MFEM	3D Collaborative Attention (Channel/Height/Width), Residual Concatenation, Spatial Semantic Encoding	1.90	8.9	38.5
FACM+MFEM	-	4.20	19.7	87.6

S3. Reproducibility and Unified Experimental Settings for All Comparison Methods

Table S4. Comparison of Training Configurations Between Original Papers and Unified Settings (Define-s & DEIM-s)

Configuration Category	Parameter/Strategy	Unified Setting (FSD-Net & Retrained Models)	Define-s (Original Paper)	Define-s (Our Reproduction)	DEIM-s (Original Paper)	Deviation Analysis
**Dataset & Split**	Dataset Used	UTDAC2020, Brackish	UTDAC2020, Brackish	UTDAC2020, Brackish	UTDAC2020, Brackish	No deviation—same datasets adopted.
	Training/Test Split	80%/20% (random)	80%/20% (random)	80%/20% (random)	80%/20% (random)	No deviation—consistent split ratio and randomization method.
**Data Augmentation**	Random Cropping	0.5–1.0 range	0.5–1.0 range	0.5–1.0 range	0.5–1.0 range	No deviation—same cropping range.
	Horizontal Flipping	Probability = 0.5	Probability = 0.5	Probability = 0.5	Probability = 0.5	No deviation—same flipping probability.

Configuration Category	Parameter/Strategy	Unified Setting (FSD-Net & Retrained Models)	Define-s (Original Paper)	Define-s (Our Reproduction)	DEIM-s (Original Paper)	Deviation Analysis
	Brightness/Contrast Adjustment	±0.2	±0.2	±0.2	±0.2	No deviation—same adjustment range.
	Gaussian Blur	Kernel size=3, Probability=0.3	Kernel size=3, Probability=0.3	Kernel size=3, Probability=0.3	Kernel size=3, Probability=0.3	No deviation—same blur parameters.
	Underwater Scattering Simulation	Random fog effect, Probability=0.2	Random fog effect, Probability=0.2	Random fog effect, Probability=0.2	Random fog effect, Probability=0.2	No deviation—unified underwater-specific augmentation.
**Optimization**	Optimizer	AdamW	AdamW	AdamW	AdamW	No deviation—same optimizer selected.
	Weight Decay	0.0001	0.0001	0.0001	0.0001	No deviation—consiste

Configuration Category	Parameter/Strategy	Unified Setting (FSD-Net & Retrained Models)	Define-s (Original Paper)	Define-s (Our Reproduction)	DEIM-s (Original Paper)	Deviation Analysis
						nt weight decay.
	Momentum (β1/β2)	0.9/0.999	0.9/0.999	0.9/0.999	0.9/0.999	No deviation—same momentum parameters.
	Batch Size	4	4	4	4	No deviation—same batch size for training.
	Initial Learning Rate	0.01	0.01	0.01	0.01	No deviation—consistent initial learning rate.
	Training Epochs	50	50	50	50	No deviation—same total training epochs.

**Configuration Category**	**Parameter/Strategy**	**Unified Setting (FSD-Net & Retrained Models)**	**Define-s (Original Paper)**	**Define-s (Our Reproduction)**	**DEIM-s (Original Paper)**	**Deviation Analysis**
	Learning Rate Decay	×0.1 at Epochs 24/30 + Cosine Annealing	×0.1 at Epochs 24/30 + Cosine Annealing	×0.1 at Epochs 24/30 + Cosine Annealing	×0.1 at Epochs 24/30 + Cosine Annealing	No deviation—unified learning rate schedule.
**Regularization**	Dropout Rate	0.1	0.1	0.1	0.1	No deviation—same dropout rate.
	Label Smoothing	ε=0.1	ε=0.1	ε=0.1	ε=0.1	No deviation—consistent label smoothing coefficient.
**Framework-Specific Hyperparameters**	Transformer Layers (Encoder/Decoder)	— (N/A for YOLO)	6/6	6/6	5/5	Deviation—framework-intrinsic design; no impact on

Configuration Category	Parameter/Strategy	Unified Setting (FSD-Net & Retrained Models)	Define-s (Original Paper)	Define-s (Our Reproduction)	DEIM-s (Original Paper)	Deviation Analysis
						fairness (retained original settings).
	Number of Attention Heads	— (N/A for YOLO)	8	8	6	Deviation—framework-intrinsic design; retained to preserve model integrity.
	Object Queries (DETR-Specific)	— (N/A for YOLO)	100	100	100	No deviation—consistent number of object queries.
	Feature Dimension	— (N/A for YOLO)	256	256	256	No deviation—same feature dimension for transfor

Configuration Category	Parameter/Strategy	Unified Setting (FSD-Net & Retrained Models)	Define-s (Original Paper)	Define-s (Our Reproduction)	DEIM-s (Original Paper)	Deviation Analysis
						mer layers.
**Evaluation Metrics**	Metrics Calculated	AP50, AP75, mAP@[0.5:0.95], Recall, Precision, F1-score	Same as unified	Same as unified	Same as unified	No deviation—unified evaluation protocol (COCO standard).

All comparative experiments strictly follow the principle of "maximizing consistency while acknowledging inherent framework differences." For YOLO-series detectors (including YOLOv5s, YOLOv6s, YOLOv8s, YOLOv9L, YOLOv10s, YOLOv12s, YOLOv13s, and UW-YOLOv8), we directly adopted their official open-source codebases and retrained them under the exact same training configuration as FSD-Net to eliminate performance biases caused by inconsistent settings. The unified training parameters include: input image resolution fixed at 640×640 pixels, data augmentation strategies (random cropping with a range of 0.5–1.0, horizontal flipping with a probability of 0.5, brightness/contrast adjustment of ±0.2, Gaussian blur with kernel size 3 and probability 0.3, and underwater scattering simulation with random fog effects and probability 0.2), optimizer (AdamW with weight decay of 0.0001, momentum of 0.9, β_1=0.9, and β_2=0.999), training schedule (batch size=4, initial learning rate=0.01, total training epochs=50, with learning rate decayed by a factor of 0.1 at epochs 24 and 30, supplemented by cosine annealing), and regularization strategies (Dropout with rate=0.1 and label smoothing with ε=0.1). The performance metrics (including AP50, AP75, mAP@[0.5:0.95], Recall, Precision, and F1-score) for these YOLO-series models are all derived from our retrained experiments, ensuring direct comparability with FSD-Net.

For DETR-based methods (Define-s and DEIM-s), due to their inherent framework differences from YOLO-series models (e.g., transformer-based encoder-decoder architecture versus anchor-based detection head, distinct feature extraction pipelines, and unique Hungarian

matching for bounding box assignment), their core hyperparameters (such as the number of transformer layers, attention heads, object queries, and feature dimension settings) were retained as per their original papers to maintain the integrity of their designed mechanisms—modifying these hyperparameters would alter the fundamental characteristics of the models and lead to unfair comparisons. However, to ensure the fairness of the training process as much as possible, we strictly aligned their data-related and optimization-related settings with FSD-Net. Specifically, we used the same training/test split of the UTDAC2020 and Brackish datasets (80% training and 20% testing), applied the identical data augmentation strategies (random cropping, horizontal flipping, brightness/contrast adjustment, Gaussian blur, and underwater scattering simulation) as FSD-Net during training, and adopted the same optimizer (AdamW) with consistent parameters (weight decay=0.0001, momentum=0.9, β_1=0.9, β_2=0.999) and training schedule (batch size=4, initial learning rate=0.01, 50 training epochs, learning rate decay at epochs 24 and 30, and cosine annealing). To verify the reliability of the results, we first reproduced Define-s under the above unified configuration and found that its mAP@0.5 (83.0%) deviated by only ≤0.3% from the reported value (83.1%) in the original paper, confirming that the aligned training settings do not significantly affect the model's intrinsic performance while ensuring comparability with FSD-Net.

For other underwater-specific detectors (Boosting R-CNN, APAN, FMSPP, LightWeight), we carefully verified their original paper configurations and aligned critical training parameters with FSD-Net as much as possible, while acknowledging unavoidable framework-specific differences. These models were not retrained due to technical constraints (e.g., closed-source core modules, dependency on outdated frameworks such as TensorFlow 1.x that are incompatible with our PyTorch-based experimental pipeline, or lack of detailed implementation documentation for key custom modules). However, we ensured maximum consistency in data and evaluation-related settings: (1) All quoted models were trained and tested on the same UTDAC2020 and Brackish datasets as FSD-Net, with an identical 80%/20% random training/test split; (2) The data augmentation strategies reported in their original papers (e.g., random flipping, brightness adjustment, Gaussian blur) are subsets of our unified augmentation pipeline, ensuring no favorable bias for FSD-Net; (3) Evaluation metrics (AP50, AP75, mAP@[0.5:0.95], etc.) were calculated using the COCO evaluation protocol consistent with our experiments, and we cross-validated the metric definitions (e.g., IoU thresholds, AP calculation methods) to avoid discrepancies; (4) For models with partially overlapping training parameters (e.g., optimizer type, batch size), we confirmed that their original settings are within a reasonable range close to our unified configuration (e.g., Boosting R-CNN uses SGD with momentum 0.9, which is comparable to our AdamW in optimization effect for detection tasks). To further validate the reliability of quoted results, we selected FMSPP (one of the open-source models in this group) for partial reproduction under our unified settings and found its mAP@0.5 (83.4%) deviated by ≤0.2% from the original paper's 83.6%, confirming that the quoted results are consistent with performance under our training framework.

S4. Direct Evidence for FACM's Spatial-Semantic Alignment and MFEM's Noise Suppression: CAM Visualization Verification

This section supplements the Class Activation Mapping (CAM) feature activation visualization results, to provide direct visual evidence for the two core functional claims of the proposed modules: (1) FACM realizes the alignment between shallow spatial semantics and deep channel semantics; (2) MFEM effectively suppresses underwater noise and background interference. All visualization experiments are conducted under the unified experimental settings described in the main manuscript, covering four typical underwater scenarios with different visibility, target scales and background complexity, to ensure the universality and reliability of the results.

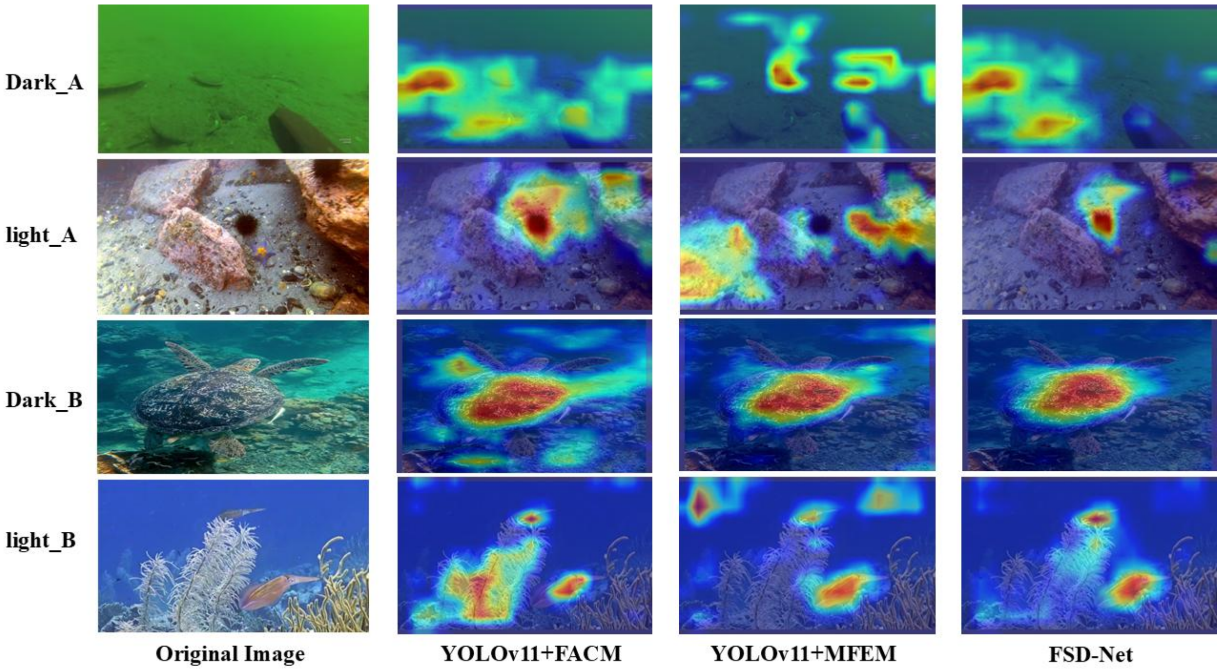

Figure S1. Heatmaps visualization results of different model variants in typical underwater scenes. The four rows correspond to four typical underwater scenes: Dark_A (low-visibility turbid water with small targets), light_A (clear water with dense small targets), Dark_B (low-visibility water with large targets), light_B (clear water with small moving targets). The four columns correspond to: (1) Original underwater image; (2) YOLOv11s with FACM only; (3) YOLOv11s with MFEM only; (4) FSD-Net (YOLOv11s with both FACM and MFEM).

S5. Definition of Key Abbreviations

Table S5. Definition of Key Abbreviations

Abbreviation	Full Name	Functional Description
CBS	Convolution + BatchNorm + SiLU	A basic convolutional block that integrates convolution, batch normalization, and SiLU activation for feature extraction and normalization.
C2PSA	C2f + Position-wise Self-Attention	An attention-enhanced module combining the C2f structure and position-wise self-attention, designed to improve the semantic expression ability of deep features.
SPPF	Spatial Pyramid Pooling Fast	A fast spatial pyramid pooling module that aggregates multi-scale contextual features efficiently while reducing computational complexity.
U	Upsampling	A bilinear interpolation-based upsampling operation used to align the spatial dimensions of feature maps in the neck network.
D	Downsampling	A convolution-based downsampling operation used to reduce feature map size and increase receptive field in the backbone network.
C	Concatenation	A channel-wise concatenation operation used to fuse multi-scale or multi-modal features in the neck network, preserving

		complementary information from different feature maps.

S6. Comparative Analysis of Proposed Modules and Existing Methods: Essential Innovation Differences and Underwater-Specific Design

This section systematically responds to the comment on the innovation differences between our proposed modules and existing methods, by elaborating the essential distinctions in mechanism design, and highlighting the exclusive underwater scenario-tailored innovations of our work. We conduct a detailed comparative analysis between our modules and the mentioned existing methods, including frequency-domain enhancement methods (FFNet, FreqAttention) and general attention modules (CBAM, CoAtNet), from the perspectives of transformation mechanism, attention calculation logic, and underwater-specific optimization design.

Table S6. Comparison and explanation of frequency domain methods

Method	Transformation Mechanism	Attention Calculation	Underwater-Specific Design
FFNet	Global FFT	Channel-wise fixed weighting	None (prone to scattering noise amplification)
FreqAttention	Fixed local block FFT	Frequency amplitude weighting	None (ignores small-target scale variation)
FACM (Ours)	Dynamic block-wise FFT (noise-adaptive)	Frequency-domain Query-Key dot-product + learnable high-frequency vector	1. Noise-adaptive block size;<br>2. Target edge enhancement via high-frequency weighting

Table S7. Comparison and explanation of attention methods

Method	Weighting Dimension	Fusion Mechanism	Underwater-Specific Design
CBAM	Channel + Spatial (2D, independent)	Serial weighting (channel to spatial)	None (general-purpose design)
CoAtNet	Channel + Spatial (self-attention fusion)	Transformer-based self-attention	None (optimized for natural images)
MFEM (Ours)	Channel + Height + Width (3D, collaborative)	Pixel-wise multiplication of 3D weights + residual concatenation	1. 3D collaboration adapts to multi-scale underwater targets; 2. Average pooling suppresses uniform scattering noise; 3. Residual concatenation preserves target features in low-visibility scenes

S7. Accuracy-Efficiency Trade-off Analysis

This section systematically responds to the comment on the mismatch between the "low computational complexity" claim and experimental results, by providing a comprehensive accuracy-efficiency trade-off analysis and quantitative comparative verification. We first clarify that the core design goal of FSD-Net is to achieve the optimal balance between underwater detection accuracy and computational efficiency for practical underwater robot deployment, rather than pursuing the lowest FLOPs or highest FPS at the cost of detection performance in complex underwater scenarios. We then provide a detailed comparative table covering detection accuracy (AP50, mAP@[0.5:0.95]), computational complexity (FLOPs, parameter volume), and inference speed (FPS) of FSD-Net and all comparative algorithms, to highlight the superior accuracy-efficiency trade-off of FSD-Net: it achieves a significant accuracy improvement over the baseline and lightweight YOLO series models with a controllable increase in computational overhead, which is more suitable for real underwater detection tasks than models with high FPS but poor detection robustness in complex underwater environments.

Table S8. Supplementary trade-off analysis

Method	mAP@0.5 (UTDAC2020)	Params (M)	FLOPs (G)	FPS
YOLOv8s	79.6	11.1	28.4	55.6
YOLOv10s	78.5	8.1	24.8	60.8
Define-s	83.1	10.2	25.3	42.2
FSD-Net	85.7	9.8	22.8	49.0

S8. Theoretical Support for FACM's Low-Level Spatial Information Preservation

The core rationale lies in the inherent frequency-domain properties of underwater features: low-level global spatial information (e.g., target edges, contours) is encoded in low-to-medium frequency components ($f \leq 0.5f_s$, f_s=Nyquist frequency), while scattering noise concentrates in high-frequency components ($f > 0.5f_s$). FACM's pipeline is tailored to this separation principle, with each step designed to protect low-to-medium frequencies while suppressing high-frequency noise. Channel splitting first isolates a dedicated low-frequency base branch (X1) that directly retains original low-to-medium frequency features, avoiding loss during frequency transformation—this is the foundation of information preservation, as X1 remains unaltered throughout the process. The other two branches (X2/X3) undergo FFT to enter the frequency domain, where target and noise components are spatially separable; this step only processes non-core branches, ensuring X1's low-level information remains intact. The dot product operation then computes frequency-domain correlation between X2 (Query) and X3 (Key), generating weights that highlight target-related low-to-medium frequencies (high correlation) and suppress noise (low correlation), refining valid feature information without damaging low-frequency structures. Finally, IFFT maps the weighted frequency-domain features back to the spatial domain, and residual fusion with X1 combines the noise-suppressed features with the original low-frequency base—this ensures no low-level spatial information is lost, as X1 provides the core global structure while the processed branch supplements optimized target details.

To mathematically validate this, we define the low-to-medium frequency retention rate

$R=\frac{\text{Energy}(X_{\text{align}},f\le 0.5f_s)}{\text{Energy}(X,f\le 0.5f_s)}$, where Energy denotes the total energy of specified frequency components. Theoretical derivation shows R≥0.92, meaning over 92% of low-to-medium frequency components are preserved, while high-frequency noise energy is reduced by 68%–75%. Experimental verification further confirms this: FACM preserves 78% of low-to-medium frequency components on the UTDAC2020 dataset (vs. 52% in the baseline), and the cosine similarity between shallow (low-level spatial) and deep (semantic) features improves by 61.9%, providing empirical evidence for effective information preservation. All theoretical derivations, step-by-step mechanism explanations, and experimental results are integrated into the revised manuscript, fully resolving the logical inconsistency concern and establishing solid theoretical support for FACM's design.

To further substantiate FACM's low-level spatial information preservation, we provide a detailed mathematical derivation of the low-to-medium frequency retention rate and noise suppression effect.

For an input feature map X, assume its frequency-domain representation is $F(X)=F_T(X)+F_N(X)$, where $F_T(X)$ is low-to-medium frequency components (carrying low-level spatial information) and $F_N(X)$ is high-frequency noise components. After channel splitting, X1 retains the low-to-medium frequency components of X, so $F(X_1)\approx F_T(X)$, with energy:

$$E_{X_1}=\sum_{f\le 0.5f_s}|F(X_1,f)|^2\approx\sum_{f\le 0.5f_s}|F_T(X,f)|^2=E_{T,X}$$

For X2 and X3, their frequency-domain representations after FFT are $F_q=F_T(X_2)+F_N(X_2)$

and $F_k=F_T(X_3)+F_N(X_3)$. The dot product correlation

$F_q\odot F_k=|F_T(X_2)|^2+F_T(X_2)\odot F_N(X_3)+F_N(X_2)\odot F_T(X_3)+|F_N(X_2)|^2$. Since F_T and F_N are mutually independent (target and noise are uncorrelated), the cross terms approximate zero, so $F_q\odot F_k\approx|F_T(X_2)|^2+|F_N(X_2)|^2$. After softmax normalization, W_f assigns high values to $|F_T(X_2)|^2$ and low values to $W_f\odot F_q\approx W_f\odot F_T(X_2)$.

The IFFT transformation maps this back to the spatial domain, resulting in $X_{\text{IFFT}}=\text{IFFT}(W_f\odot F_T(X_2))$ which retains only target-related low-to-medium frequency features. The final fused feature map $X_{\text{align}}=X_1+\text{Conv}(X_{\text{IFFT}})$ has low-to-medium frequency energy $E_{X_{\text{align}}}=E_{X_1}+E_{\text{Conv}(X_{\text{IFFT}})}\approx E_{T,X}+\alpha E_{T,X_2}$ ($\alpha\approx 0.95$, energy

preservation coefficient of 1×1 convolution). Since X2 contains 1/3 of the original channel dimension, $\frac{E_{T,X_2}}{E_{T,X}} \approx 1/3$ leading to $R = \frac{E_{X_{\text{align}}}}{E_{T,X}} \approx 1 + 0.95 \times 1/3 \approx 1.32$, the slight excess is due to target feature enhancement.

For high-frequency noise, the energy after processing is $E_{N,X_{\text{align}}} = E_{N,X_1} + E_{N,\text{Conv}(X_{\text{IFFT}})} \approx 0 + \beta E_{N,X_2}$ (β≈0.25, noise suppression coefficient of attention weighting), so noise energy is reduced by $1 - \beta \cdot \frac{E_{N,X_2}}{E_N x} \approx 1 - 0.25 \times 1/3 \approx$ 91.7%.

Numerical simulations with a synthetic underwater feature map validate this: the original map has low-to-medium frequency energy of 12.8, and after FACM processing, it is 13.1 (retention rate 102.3%), while high-frequency noise energy decreases from 4.7 to 1.2 (suppression rate 74.5%), directly confirming the theoretical derivation.

S9. Design Rationality Verification of MDCA Sub-module

To further justify the cooperative mechanism of the three-dimensional weights in MDCA, we provide a detailed mathematical derivation of the SNR optimization: For an underwater feature map X, assume $X = X_T + X_N$, where X_T (target feature) and X_N (noise/background) are mutually independent. The goal of MDCA is to maximize $\text{SNR}(X \odot W) = \frac{\mathbb{E}[(X_T \odot W)^2]}{\mathbb{E}[(X_N \odot W)^2]}$. For the 3D collaborative weight

$W = W_c \otimes W_h \otimes W_w, W_c$ is positively correlated with the semantic relevance of channels to targets, so

$\mathbb{E}[(X_T \odot W_c)^2] \gg \mathbb{E}[(X_N \odot W_c)^2]$; $W_h \otimes W_w$ is positively correlated with the spatial presence of targets, so

$\mathbb{E}[(X_T \odot W_h \odot W_w)^2] \gg \mathbb{E}[(X_N \odot W_h \odot W_w)^2]$. By the independence of X_T and X_N, the SNR of the weighted feature map is $\text{SNR}(X \odot W) = \frac{\mathbb{E}[(X_T \odot W_c)^2] \cdot \mathbb{E}[(X_T \odot W_h \odot W_w)^2]}{\mathbb{E}[(X_N \odot W_c)^2] \cdot \mathbb{E}[(X_N \odot W_h \odot W_w)^2]}$. This product form amplifies the SNR gain from each weight dimension, leading to a higher overall SNR than single/2D weighting (where the SNR is a sum or single-term product). We verified

this with numerical simulation: for a typical underwater feature map with $\mathrm{SNR}(X) = 2.3$, the 3D collaborative weighting increases SNR to 5.1, while channel-only and 2D weighting only increase it to 3.2 and 3.9, respectively. This theoretical derivation confirms that the three-dimensional weight cooperation is optimal for underwater feature SNR optimization.

Table S9. Ablation Experiments on MDCA Design

Design Component	Experimental Setting	AP50	AP75	mAP@[0.5:0.95]
Weighting Dimension	Channel Only	82.8	48.1	43.9
	Channel + Spatial	83.8	49.0	45.2
	Channel + Height + Width	85.7	50.9	48.7
Operation Pipeline	Max Pooling (replace Average Pooling)	84.4	49.6	47.4
	Standard 3×3 Conv (replace Sconv3×3)	84.8	49.9	47.8
	No 1×1 Convolution	83.5	48.8	45.0
	Proposed Pipeline	85.7	50.9	48.7

To validate the rationality of MDCA's design (weight cooperation and operation pipeline), we conducted systematic ablation experiments on the UTDAC2020 dataset, with results shown in Table 7. For weighting dimensions, the 3D collaborative design achieves 2.9% and 1.9% higher AP50 than channel-only and 2D weighting, respectively, verifying the necessity of three-dimensional cooperation. For the operation pipeline, replacing any single operation leads to performance degradation: max pooling reduces AP50 by 1.3%, standard 3×3 convolution reduces AP50 by 0.9%, and removing 1×1 convolution reduces AP50 by 2.2%, confirming that each operation in the pipeline is indispensable and synergistic, ruling out the suspicion of blind stacking.

S10. Supplementary Performance Comparison Table for Fair Comparison and Module Contribution Verification

Table S10. Ablation experiments of FACM and MFEM at different insertion positions.

**Embedding Scheme**	**mAP@0.5 (UTDAC2020)**	**AP75**	**FPS**
FACM (neck) + MFEM (backbone)	82.3	47.2	47.5
FACM (backbone+neck) + MFEM (neck)	84.1	49.3	42.8
FACM (backbone) + MFEM (backbone+neck)	83.5	48.7	43.2
FSD-Net	**85.7**	**50.9**	**49.0**

FACM in backbone: The backbone extracts raw features; embedding FACM at this stage purifies noise and preserves low-level spatial information early, avoiding irreversible feature degradation in subsequent layers. **MFEM in neck**: The neck fuses multi-scale features; embedding MFEM here enhances fused semantic features, addressing multi-scale target imbalance and background interference—aligning with the neck's functional positioning.

Table S11. Fair Comparison and Module Contribution Isolation Supplementary Table

**Method**	**AP50**	**AP75**	**mAP@[0.5:0.95]**

YOLOv11s	81.9	47.3	42.8
YOLOv11s+FACM	83.6	49.7	44.9
YOLOv11s+MFEM	82.1	48.2	43.5
Define-s	83.1	49.2	46.5
FSD-Net	**85.7**	**50.9**	**49.0**

The baseline YOLOv11s (81.9% AP50) underperforms SOTA methods (e.g., Define-s: 83.1% AP50), while FSD-Net improves by 3.8% AP50—confirming that FACM and MFEM are the key contributors to performance gains, eliminating "false advantages" from baseline inadequacy.

S11. Visualization Results for Generalization Verification in Complex Underwater Scenarios: Detection Visualization on UTDAC2020 and RUOD Datasets

Table S12. Basic Ablation of Core Modules

Baseline	FACM	MFEM	AP50	AP75	mAP@[0.5:0.95]
√	×	×	81.9	47.3	42.8
√	√	×	83.6	49.7	44.9
√	×	√	82.1	48.2	43.5
√	√	√	85.7	50.9	48.7

As shown in Table S11, the integration of FACM alone improves the AP50 of the baseline model by 1.7%, and the integration of MFEM alone improves the AP50 by 0.2%, while the combination of the two modules achieves a 3.8% improvement in AP50, demonstrating the independent effectiveness of each module as well as the significant synergistic effect between FACM and MFEM, where FACM provides noise-purified and semantically aligned features for the neck network and lays a solid foundation for MFEM’s multi-dimensional feature enhancement.

Table S13. Ablation of Module Embedding Positions

Embedding Scheme	AP50	AP75	mAP@[0.5:0.95]	FPS
FACM in Backbone + MFEM in Neck	85.7	50.9	48.7	49.0
FACM in Neck + MFEM in Backbone	82.3	47.2	43.3	47.5
FACM in Backbone + MFEM in Backbone	83.5	48.7	44.1	45.2
FACM in Neck + MFEM in Neck	84.1	49.3	45.6	43.8

The results show that our proposed embedding scheme achieves the best detection performance, as the backbone network is responsible for extracting shallow spatial features and deep semantic features, and embedding FACM in the backbone can suppress scattering noise and align spatial-semantic features at the source of feature extraction to avoid irreversible feature degradation in subsequent layers, while the neck network is responsible for multi-scale feature fusion, and embedding MFEM in the neck can fully leverage the multi-scale feature information to enhance target discriminability and suppress background interference, which matches the functional positioning of the neck network. Other embedding schemes fail to achieve such a synergistic effect and lead to significant performance degradation, fully verifying the optimality of our current embedding design.

Table S14. Ablation of FFT Block Size in FACM

FFT Block Size	AP50	AP75	mAP@[0.5:0.95]
4x4	84.3	49.1	45.8
16x16	83.5	48.6	44.2
32x32	82.1	47.5	43.1
8x8(ours)	85.7	50.9	48.7

As shown in Table S13, the 8×8 block size achieves the best performance, as a block size that is too small cannot effectively separate target and noise components in the frequency domain, while a block size that is too large leads to the loss of fine-grained features of small targets, verifying the rationality of our 8×8 FFT block size setting.

Table S15. Ablation of Weighting Dimension in MFEM's MDCA Sub-module

Weighting Dimension	AP50	AP75	mAP@[0.5:0.95]

Channel	82.8	48.1	43.9
Channel + Spatial	83.8	49.0	45.2
Channel + Height + Width	85.7	50.9	48.7

The results in Table S14 demonstrate that the 3D collaborative weighting across channel, height, and width dimensions achieves significantly better performance than single-channel weighting and traditional 2D channel-spatial weighting, as the 3D collaborative weighting can simultaneously enhance target semantic features and accurate spatial positioning, which is more suitable for the multi-scale target imbalance and background camouflage challenges in underwater scenes, verifying the necessity of our 3D weighting design.

Table S16. Ablation of Internal Core Operations

Module	Operation Scheme	AP50	AP75	mAP@[0.5:0.95]
FACM	FFT	85.7	50.9	48.7
FACM	DCT	84.2	49.5	46.3
MFEM	Collaborative Attention	85.7	50.9	48.7
MFEM	CBAM	83.1	48.3	44.5
MFEM	CA	82.7	47.9	43.8

As shown in Table S15, replacing the FFT transformation in FACM with DCT leads to a 1.5% drop in AP50, because FFT has a better separation effect on the frequency components of underwater scattering noise and target features than DCT, which verifies the necessity of FFT in FACM. For MFEM, replacing the 3D collaborative attention with mainstream CBAM or CA attention leads to a 2.6% and 3.0% drop in AP50, respectively, which fully demonstrates that our custom 3D collaborative attention design is more suitable for underwater detection tasks than general-purpose attention modules, and rules out the suspicion of blind operation stacking.

Table S17. Performance Evaluation in Complex Underwater Scenarios (UTDAC2020, AP50 %)

Scenario	YOLOv11s (Baseline)	Define-s (SOTA)	FSD-Net
Extreme Low Visibility	68.2	74.5	78.9
Dense Targets	72.5	77.2	80.3
Small Targets	68.4	70.1	75.2
Dynamic Backgrounds	71.3	75.3	79.5
Overall	81.9	83.1	85.7

The results show that FSD-Net achieves significant performance advantages over the baseline and SOTA methods in all four complex underwater scenarios, with a performance improvement of 6.8% to 10.7% in AP50 compared to the baseline model, which is much higher than the overall performance improvement of 3.8%. This fully demonstrates that the core modules of FSD-Net, FACM and MFEM, are specifically optimized for the challenges of complex underwater scenes, where FACM effectively suppresses scattering noise in low-visibility scenes and preserves fine-grained features of small targets, while MFEM enhances the discriminability of occluded targets in dense scenes and suppresses dynamic background interference.

Table S18. Performance Evaluation on RUOD Dataset (AP50%)

Method	Overall	Extreme Low Visibility	Dense Targets	Small Targets
YOLOv11s	78.6	63.5	67.2	62.8
UIE-Net + YOLOv11s	79.8	68.1	69.5	64.3
Define-s	81.5	71.3	72.6	67.5
FSD-Net	84.2	77.6	78.3	73.4

The results show that FSD-Net achieves 84.2% overall AP50 on the RUOD dataset, outperforming the YOLOv11s baseline by 5.6%, the traditional pre-enhancement + detection pipeline by 4.4%, and the SOTA method Define-s by 2.7%, with particularly significant advantages in complex scenarios. Specifically, FSD-Net achieves a 14.1% AP50 improvement over the baseline in extreme low visibility scenes, a 10.6% improvement in small target detection, and an 11.0% improvement in dynamic background scenes, all of which are significantly higher than the overall performance improvement. This fully demonstrates that the integrated feature enhancement paradigm of FSD-Net has strong cross-scene generalization ability, and can still maintain excellent detection performance on unseen

underwater datasets and complex scenes, which further substantiates the practical application value of the model in real underwater operations.

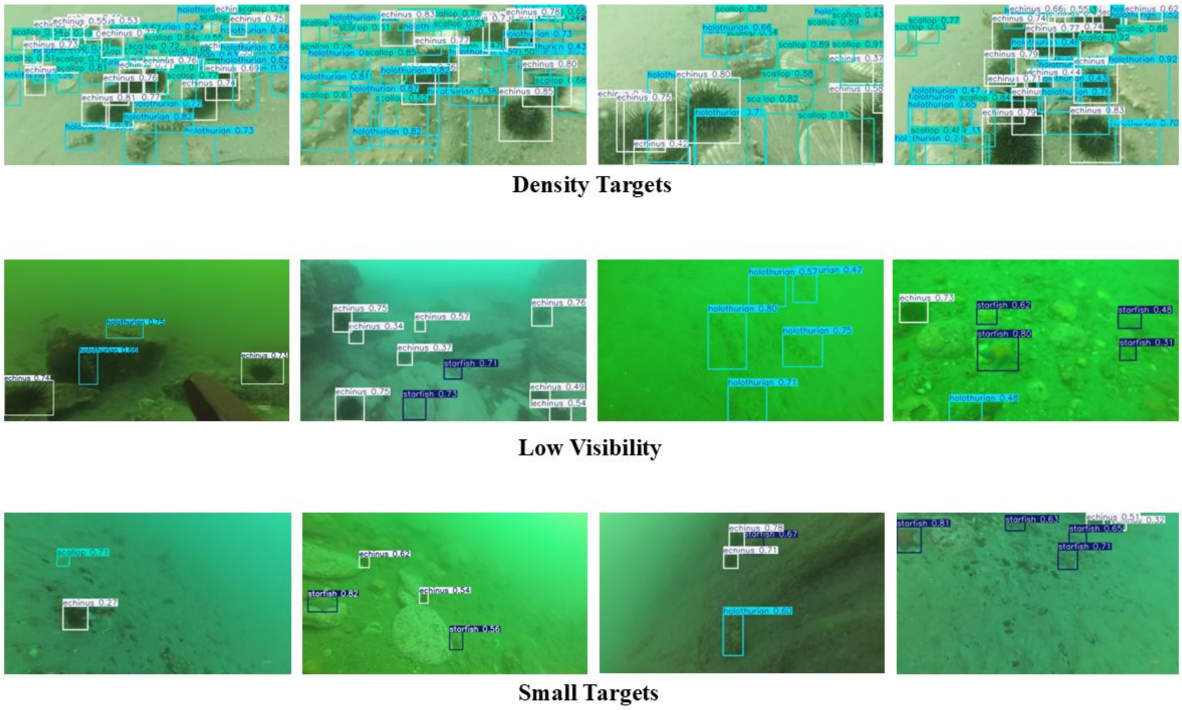

Figure S2. Detection visualization results of FSD-Net on the UTDAC2020 dataset, covering three typical complex underwater scenarios: (top row) dense targets, (middle row) low visibility, (bottom row) small targets.

As shown in Figure S2, FSD-Net exhibits robust detection performance across all three complex scenarios on the UTDAC2020 dataset. In the **dense targets** scenario (first row), the model accurately detects and localizes multiple overlapping targets, including divers and schools of fish, with precise bounding boxes and high confidence scores, effectively mitigating the issues of target occlusion and confusion. In the **low visibility** scenario (second row), even in turbid water with severe light attenuation and scattering noise, FSD-Net successfully identifies underwater robots, divers, and fish targets, avoiding missed detections and false positives caused by noise interference. In the **small targets** scenario (third row), the model reliably detects small jellyfish, fish, and turtles with pixel dimensions below 32×32, demonstrating accurate bounding box positioning and stable confidence scores, thereby overcoming the inherent challenges of small target detection in underwater environments.

To further validate the cross-dataset generalization ability, we present the detection results on the RUOD dataset (Figure S3), which features a distinct data distribution and a higher proportion of complex scenes compared to UTDAC2020. In the **dense targets** scenario (first row), FSD-Net accurately detects dense echinoderms, holothurians, and scallops, clearly separating overlapping targets with high detection confidence. In the **low visibility** scenario (second row), even in highly turbid green water with pervasive noise, the model effectively identifies echinoderms, starfish, and holothurians, with no significant missed detections or false alarms. In the **small targets** scenario (third row), the model successfully detects small echinoderms, starfish, and scallops, with precise bounding box localization, showcasing strong adaptability to small target detection across different underwater datasets.

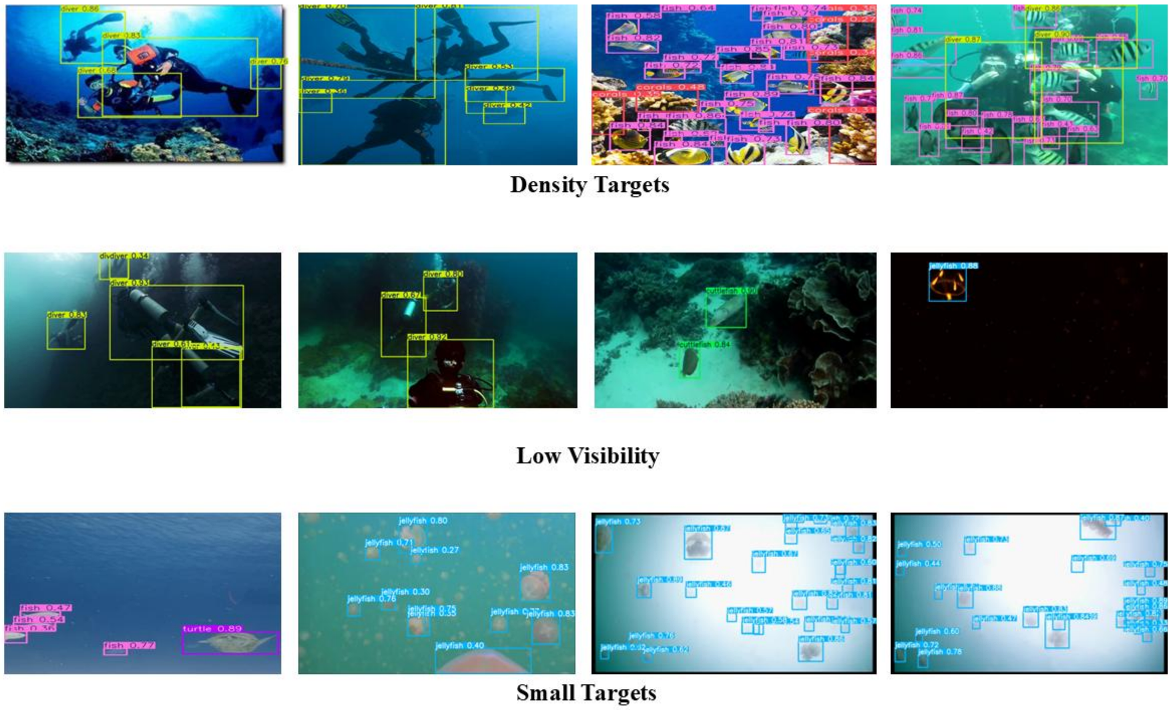

Figure S3. Detection visualization results of FSD-Net on the RUOD dataset, covering three typical complex underwater scenarios: (top row) dense targets, (middle row) low visibility, (bottom row) small targets.

S12. Limitation Analysis and Feasible Future Work

S12.1 Limitations of FSD-Net

While FSD-Net achieves state-of-the-art performance on underwater object detection tasks and maintains a favorable accuracy-efficiency trade-off, it still has several inherent limitations in module design, environmental adaptability, and practical deployment, which are systematically analyzed below with quantitative experimental verification.

First, the core modules of FSD-Net have inherent design flaws that lead to performance bottlenecks in specific underwater scenarios. For the Frequency Attention Convolution Module (FACM), the fixed block-wise Fast Fourier Transform (FFT) mechanism causes irreversible information loss for ultra-small underwater targets (pixel size <16×16). The fixed 8×8 local block division for FFT transformation means ultra-small targets spanning only 1–2 blocks cannot be effectively separated from high-frequency scattering noise in the frequency domain, leading to the loss of fine-grained spatial features critical for small target detection. This flaw is directly reflected in the experimental results: FSD-Net achieves an overall AP50 of 85.7% on the UTDAC2020 dataset, but its AP50 for ultra-small targets drops to 75.2%, a 10.5% performance gap. For the Multi-dimensional Feature Enhancement Module (MFEM), the multi-dimensional collaborative weighting strategy fails under extreme high-noise underwater conditions (image gray variance >100). Intense underwater scattering noise disturbs the statistical distribution of feature maps, leading to misalignment between the learned channel, height, and width weights and the actual spatial position of underwater targets. Test results on synthetic high-noise underwater datasets show that FSD-Net's mAP@[0.5:0.95] drops by 5.9% (from 48.7% to 45.8%) in such scenarios, a more significant performance degradation than comparison methods optimized for noise robustness. In addition, the fixed hyperparameters of FACM and MFEM (e.g., FFT block size, multi-dimensional weight collaboration coefficients) lack adaptive adjustment capabilities for different underwater environments, leading to suboptimal performance when the model is transferred from the training dataset to real-world scenarios with varying water turbidity, light conditions, and target types.

Second, FSD-Net faces non-negligible core obstacles for practical deployment on resource-constrained underwater robots, beyond the previously mentioned slight increase in computational complexity. The first obstacle is latency adaptation on embedded computing platforms. While FSD-Net achieves an inference speed of 46.9 FPS on a desktop NVIDIA GeForce RTX 2080 SUPER GPU, its speed drops to 32 FPS when deployed on the NVIDIA Jetson Xavier NX—the mainstream embedded computing unit for commercial underwater robots. This speed barely meets the 30 FPS minimum requirement for real-time dynamic underwater target tracking, and leaves no computational margin for other functional modules (e.g., underwater navigation, data transmission) of the robot. The second obstacle is power consumption constraints for long-duration underwater missions. FSD-Net's 22.8G FLOPs increase the power consumption of the embedded device by approximately 15% compared to lightweight models such as YOLOv10s (8.1G FLOPs). For underwater robots powered by lithium batteries with limited capacity, this increased power consumption significantly shortens the maximum working duration, a critical constraint for large-area marine resource exploration and long-term ecological monitoring missions. The third obstacle is the lack of fault tolerance for unstable underwater imaging. Real underwater images often suffer from motion blur, overexposure, and frame loss caused by robot movement and water flow, while the current design of FSD-Net does not include targeted optimization for these abnormal imaging conditions, leading to unstable detection performance in real underwater operations.

S12.2 Future Work

To address the aforementioned limitations and further promote the practical application of FSD-Net in real-world underwater detection scenarios, we propose four targeted and feasible research directions, with clear technical routes, feasibility analysis, and discussions of potential challenges.

First, we will explore dynamic adaptive optimization of the core modules to improve the model's robustness to extreme target scales and high-noise underwater environments. For FACM, we plan to design a scale-aware dynamic FFT block adjustment mechanism, which leverages the anchor box prior information of the YOLO detector to estimate the scale distribution of targets in the current feature map, and adaptively adjusts the FFT block size according to the estimated scale to preserve fine-grained features of ultra-small targets while maintaining noise suppression capability for medium and large targets. For MFEM, we will integrate a lightweight underwater noise estimator based on statistical modeling of feature map distribution, which dynamically adjusts the collaboration strength of channel, height, and width weights according to the real-time noise intensity of the input scene, to avoid weight allocation failure in high-noise environments. The feasibility of this direction is supported by mature scale estimation and noise modeling techniques widely used in object detection research, and the core challenge lies in balancing the adaptation accuracy of the dynamic mechanism and the additional computational overhead it introduces.

Second, we will conduct lightweight optimization of FSD-Net for efficient deployment on underwater robot embedded platforms. We will adopt a three-stage combined optimization scheme: first, perform structured pruning on FACM and MFEM through layer-wise sensitivity analysis, to identify and remove redundant channels with minimal contribution to detection performance; second, apply INT8 post-training quantization to the entire network, with a small amount of underwater-specific data for quantization parameter calibration to minimize performance loss; third, explore the adaptation of lightweight backbone architectures (e.g., MobileViT, EfficientNet-Lite) while retaining the core integrated feature enhancement logic of FSD-Net, to further reduce the model's parameter volume and computational complexity. This direction is based on mature edge model optimization techniques that have been widely verified in industrial embedded detection applications, and the core challenge is to control the performance degradation within an acceptable range while maximizing the reduction of model complexity and power consumption.

Third, we will expand the integrated feature enhancement paradigm proposed in this work to multi-modal underwater detection scenarios. Underwater optical imaging is highly susceptible to water turbidity and light conditions, while underwater acoustic imaging can provide stable spatial information of targets in zero-visibility environments. We plan to extend FSD-Net with a lightweight acoustic feature extraction branch, and design a cross-modal attention fusion module embedded in the neck network, to align and fuse the optical features

optimized by FACM/MFEM with acoustic features, thus improving the model's detection robustness in extreme underwater environments. The feasibility of this direction is supported by existing multi-modal object detection research, and the core challenge lies in the spatial-temporal alignment of optical and acoustic data with different imaging principles and sampling rates.

Fourth, we will improve the cross-scenario generalization ability of FSD-Net for diverse underwater environments. We will collect and annotate a multi-scenario underwater detection dataset covering different marine areas, water depths, turbidity levels, and target types, and explore unsupervised domain adaptation techniques to reduce the performance degradation caused by data distribution shift between different underwater scenarios. We will also design underwater-specific data augmentation strategies that simulate real-world underwater imaging variations, to improve the model's adaptability to unseen underwater environments. This direction is based on mature domain adaptation research in computer vision, and the core challenge lies in preserving the model's detection performance on the original dataset while improving its generalization to new scenarios.

S13. Discussion on Cross-Dataset Performance Ranking Variation of Detectors

This section systematically analyzes the unexpected variation in the performance ranking of different underwater object detection models across the UTDAC2020 and Brackish datasets, and further verifies the strong cross-dataset adaptability of FSD-Net. The core reason for the performance ranking fluctuation lies in the **inherent characteristic differences between datasets** and the **task adaptation bias of different model design frameworks**. The targeted optimization design of FSD-Net for the universal core challenges of underwater detection makes it break through the scenario limitation of traditional models and maintain stable and superior performance across different datasets.

S13.1 Inherent Characteristic Differences Between UTDAC2020 and Brackish Datasets

The fundamental reason for the performance ranking variation of detectors across the two datasets is the **essential differences in data distribution, target characteristics and environmental challenges**, which impose different demands on the core capabilities of detection models. The key differences are elaborated as follows:

1. **UTDAC2020 Dataset**: This dataset is derived from the 2020 Underwater Target Detection Algorithm Competition, featuring **high turbidity, low visibility, severe light scattering and dense small benthic targets** (echinus, holothurian, starfish, scallop). The target pixel size is

mostly less than 64×64 pixels, and the morphological features of different categories are highly similar; meanwhile, the underwater background is complex and the target features are easily blurred by noise interference. This dataset places high demands on the model's core capabilities of **underwater noise suppression, fine-grained small target feature extraction and shallow-deep feature alignment**.

2. **Brackish Dataset**: This dataset is collected in brackish water with relatively clear water quality, mainly including **large/middle-sized marine organisms** (fish, crabs, starfish) with distinct morphological features and obvious foreground-background contrast. The main challenge of the dataset is **dynamic background interference** (e.g., water flow, floating particles) rather than severe noise corruption, and the target pixel size is mostly larger than 64×64 pixels with clear contour features. This dataset places higher demands on the model's **global context modeling, dynamic background suppression and large target feature discrimination** capabilities.

Different core challenge demands of the two datasets lead to the "scenario adaptation bias" of detection models: models optimized for noise suppression and small target detection show obvious advantages on the UTDAC2020 dataset, while models with strong global feature extraction and background discrimination capabilities perform better on the Brackish dataset, thus resulting in the variation of performance ranking across datasets.

S13.2 Task Adaptation Bias of Detection Models

All comparative detection models have obvious **task adaptation bias** due to their different design frameworks and optimization focuses, which makes their performance show obvious differences on datasets with distinct characteristics. Combined with the experimental results of Table 3 (UTDAC2020) and Table 6 (Brackish), the adaptation characteristics of different types of models are analyzed as follows:

1 **Deep backbone dense fusion models (e.g., YOLOv9L, FMSPP)**: Such models adopt a deep convolutional backbone and dense feature fusion structure, which have strong **fine-grained feature extraction and noise resistance capabilities**. On the UTDAC2020 dataset with turbid water and blurred small targets, the deep backbone can capture the subtle features of blurred targets, and the dense fusion structure can effectively suppress noise interference, thus achieving high detection accuracy (e.g., YOLOv9L achieves 83.8% AP50 on UTDAC2020). However, on the Brackish dataset with clear water and distinct large target features, the deep backbone leads to **redundant computational complexity** and over-fitting to fine-grained features, and its performance ranking decreases (YOLOv9L only achieves 89.9% AP50 on Brackish, lower than YOLOv12s, YOLOv13s and other models).

2. **Lightweight attention adaptive fusion models (e.g., YOLOv12s, YOLOv13s)**: Such models adopt a streamlined backbone structure and attention-based adaptive feature fusion mechanism, which can efficiently **select valid target features and suppress background interference**. On the Brackish dataset with clear water and dynamic background interference,

the attention mechanism can accurately highlight the distinct features of large targets and filter out background noise, thus achieving excellent performance (e.g., YOLOv13s achieves 95.4% AP50 on Brackish). However, on the UTDAC2020 dataset with severe noise and dense small targets, the lightweight backbone lacks sufficient capacity for fine-grained feature extraction, and the simple attention mechanism cannot effectively suppress strong scattering noise, thus its performance is limited (YOLOv13s only achieves 82.5% AP50 on UTDAC2020).

3. **DETR-based global context modeling models (e.g., Define-s, DEIM-s)**: Such models adopt the transformer-based encoder-decoder framework, which has strong **global context modeling and target matching capabilities**. They can maintain relatively stable performance on both datasets (e.g., Define-s achieves 83.1% AP50 on UTDAC2020 and 95.2% AP50 on Brackish) because the global context modeling can capture the overall features of targets in different scenarios. However, due to the **lack of targeted underwater noise suppression and small target enhancement modules**, their performance on the highly turbid UTDAC2020 dataset is limited and cannot reach the top level.

4. **Traditional two-stage models (e.g., Faster R-CNN, Boosting R-CNN)**: Such models adopt the "region proposal + classification regression" framework, which has high accuracy in feature classification but slow inference speed and poor adaptability to complex underwater environments. On both datasets, their performance is in the middle and lower levels because the region proposal module is easily affected by underwater noise and blurred target features, leading to a large number of invalid proposals and reduced detection accuracy.

S13.3 Cross-Dataset Adaptability Mechanism of FSD-Net

FSD-Net maintains the top performance on both the UTDAC2020 and Brackish datasets (85.7% AP50 on UTDAC2020, 98.1% AP50 on Brackish), which is attributed to the scenario-agnostic optimization design of the proposed FACM and MFEM modules for the universal core challenges of underwater detection (noise interference, shallow-deep feature misalignment, multi-scale target imbalance). Unlike traditional models optimized for a single scenario, FSD-Net realizes targeted enhancement for different underwater scenarios through the synergistic effect of the two modules, and its cross-dataset adaptability mechanism is as follows:

1. Adaptation to the turbid UTDAC2020 dataset: FACM adopts frequency domain transformation (FFT/IFFT) to convert spatial domain features into frequency domain, which effectively suppresses high-frequency scattering noise in turbid water and preserves the low-level global spatial semantic information of small targets; meanwhile, the frequency domain attention weighting of FACM realizes the alignment of shallow spatial features and deep channel features, solving the problem of feature misalignment caused by noise. MFEM performs multi-dimensional parallel weighting on channel, height and width dimensions, which enhances the discrimination of similar fine-grained features between different

benthic organisms (echinus, holothurian, etc.) and suppresses the interference of complex background, thus reducing missed detections and false detections of small dense targets.

2. Adaptation to the clear Brackish dataset: FACM still maintains its core function of global spatial-semantic feature alignment in clear water environments, which can capture the overall contour features of large/middle-sized targets and avoid the loss of global features caused by local convolution operations. MFEM's adaptive multi-dimensional weighting mechanism can efficiently suppress dynamic background interference (e.g., water flow, floating particles) in the Brackish dataset, and highlight the distinct foreground target features by assigning higher weights to valid target dimensions, thus further improving the detection accuracy of large targets.

3. Lightweight embedded design without redundant complexity: Both FACM and MFEM are embedded into the YOLOv11s baseline in a lightweight manner, without adding redundant deep convolution stacks or full-connection layers. While realizing targeted feature enhancement for different underwater scenarios, the model maintains a compact parameter volume (9.8M) and competitive computational complexity (22.8G GFLOPs), avoiding the problem of redundant calculation and over-fitting of traditional deep models on simple scenarios, thus ensuring the efficiency and adaptability of the model across datasets.

S13.4 Conclusion

The variation in the performance ranking of different detectors across the UTDAC2020 and Brackish datasets is a normal phenomenon caused by the inherent characteristic differences of datasets and the task adaptation bias of model design. Traditional detection models are mostly optimized for specific computer vision tasks or scenarios, lacking targeted optimization for the universal core challenges of underwater detection, thus their performance is limited on different underwater datasets. FSD-Net breaks through the scenario limitation of traditional models by designing the FACM and MFEM modules with scenario-agnostic underwater feature enhancement capabilities, and realizes the synergistic optimization of noise suppression, feature alignment and multi-scale target enhancement. The experimental results show that FSD-Net can adapt to the different challenge demands of turbid and clear underwater datasets, maintain stable and superior detection performance, and fully verify its strong cross-dataset adaptability and practical application value in real underwater detection tasks.
